# A binding site for the antibiotic GE81112 in the ribosomal mRNA channel

**DOI:** 10.1128/mbio.03978-24

**Published:** 2025-10-24

**Authors:** Andreas Schedlbauer, Xu Han, Wouter van Bakel, Tatsuya Kaminishi, Borja Ochoa-Lizarralde, Idoia Iturrioz, Retina Çapuni, Ransford Parry, Ronny Zegarra, David Gil-Carton, Jorge P. López-Alonso, Kristina Barragan Sanz, Letizia Brandi, Claudio O. Gualerzi, Paola Fucini, Sean R. Connell

**Affiliations:** 1 Ribosome Structural and Functional Biology, Center for Cooperative Research in Biosciences (CIC bioGUNE), Basque Research and Technology Alliance (BRTA), Derio, Spain; 2 Precision Medicine and Metabolism Lab, Center for Cooperative Research in Biosciences (CIC bioGUNE) 568373 , Derio, Basque Country, Spain; 3 Structural Biology of Cellular Machines Laboratory, Biobizkaia Health Research Institute, Cruces University Hospital 16494, Barakaldo, Basque Country, Spain; 4 Graduate School of Medicine, Osaka University 13013, Osaka, Japan; 5 Instituto Biofisika (UPV/EHU, CSIC), University of the Basque Country, Leioa, Basque Country, Spain; 6 Department of Medicine, Faculty of Technical Medical Sciences, Western Balkans University, Tirana, Albania; 7 Research Centre for Experimental Marine Biology and Biotechnology, Plentzia Marine Station of the University of the Basque Country (PiE-UPV/EHU) 684055 , Plentzia, Basque Country, Spain; 8 Basque Resource for Electron Microscopy, Leioa, Basque Country, Spain; 9 IKERBASQUE, Basque Foundation for Science 197447, Bilbao, Basque Country, Spain; 10 Laboratory of Genetics, Department of Biosciences and Veterinary Medicine, University of Camerino 18959, Camerino, Italy; Case Western Reserve University School of Medicine, Cleveland, Ohio, USA

**Keywords:** cryo-electron microscopy, cryo-EM, antibiotic, ribosome, GE81112, initiation, IF1, IF2, IF3

## Abstract

The initiation phase is the rate-limiting step of protein synthesis (translation) and is finely regulated, making it an important drug target. In bacteria, initiation is guided by three initiation factors and involves positioning the start site on the messenger RNA within the P-site on the small ribosomal subunit (30S), where it is decoded by the initiator fMet–tRNA. This process can be efficiently inhibited by GE81112, a natural hydrophilic, noncyclic, nonribosomal tetrapeptide. It is found in nature in three structural variants (A, B, and B1 with molecular masses of 643–658 Da). Previous biochemical and structural characterization of GE81112 indicates that the primary mechanism of action of this antibiotic is to (i) prevent the initiator fMet–tRNA from binding correctly to the P-site and (ii) block conformational rearrangements in initiation factor IF3, resulting in an unlocked 30S pre*IC* state. In this study, using cryo-EM, we have determined the binding site of GE81112 in initiation complexes (3.2–3.7 Å) and on empty ribosomes (2.09 Å). This binding site is within the mRNA channel but remote from the binding site of the initiation factors and initiator fMet–tRNA. This suggests that it acts allosterically to prevent the initiator fMet–tRNA from being locked into place. The binding mode is consistent with previous biochemical studies and recent work identifying the key pharmacophores of GE81112.

**IMPORTANCE:**

This study uses high-resolution cryo-electron microscopy (cryo-EM) to reveal the precise binding site of the antibiotic GE81112 on the bacterial ribosome's 30S subunit. GE81112 targets the initiation phase of bacterial protein synthesis, specifically interacting within the mRNA channel, distant from the initiation factor and initiator tRNA-binding sites. This indicates that GE81112 acts allosterically, disrupting and preventing conformational rearrangements in IF3 and the proper positioning of the initiator tRNA, stalling the ribosome in an unlocked pre-initiation complex. The findings identify key ribosomal interactions, including conserved nucleotides in helices 23, 24, and 45, and protein S11, highlighting GE81112's unique binding mode among initiation inhibitors. This structural characterization enhances our understanding of antibiotic interference with translation initiation and provides insights to support rational design strategies for improved GE81112 derivatives.

## INTRODUCTION

In all organisms, the initiation phase is the rate-limiting step of protein synthesis (translation) and is subject to fine-tuning via post-transcriptional regulatory mechanisms (1, 2). As such, the initiation step in protein synthesis is an important drug target (3). The initiation phase is a multistep, dynamic process that begins with the formation of a 30S pre-initiation complex (pre*IC*). In the pre*IC,* the messenger RNA (mRNA), initiator-tRNA (fMet–tRNA), and three protein factors, termed initiation factors (IFs: IF1, IF2, and IF3), assemble on the small ribosomal subunit (30S subunit) to recognize the mRNA start codon (2). In this state, the fMet–tRNA is not tightly bound (4), and therefore, it is referred to as an unlocked pre*IC*. This unlocked complex undergoes a conformational change where the carboxy-terminal domain (CTD) of IF3, positioned at the top of 16S rRNA helix 44 (h44) in the pre*IC*, moves to a second position on h44 (5–7). This movement allows the fMet-tRNA to be fully accommodated in the peptidyl-tRNA binding site (P-site), locking the fMet–tRNA in place and forming a 30S initiation complex (30*IC*). The resulting 30*IC* joins the large ribosomal subunit (LSU, the 50S), releasing IF1 and IF3 to form the 70S ribosomal initiation complex (70S*IC*).

GE81112 is a specific inhibitor of this initiation process and blocks the locking step that marks the transition from the pre*IC* to the *IC* (8, 9). GE81112 is a peptide antibiotic formed by four non-proteinogenic L-amino acids, referred to as AA1-4. When purified from the producing *Streptomyces* species, GE81112 is a mixture of three congeners, termed GE81112A, GE81112B, and GE81112B1 (643–658 Da) (10). Owing to its novel chemical scaffold and potential as a lead molecule for antibiotic development, several enzymatic and chemical synthesis pathways have been established for GE81112 (11–14). This has allowed structure-activity relationship (SAR) studies to define GE81112 key pharmacophores (11, 12). For example, the work of Zwick et al. (12) highlights the importance of AA1 and AA4 for antibacterial activity. The Bauer group has also performed extensive pharmacokinetic profiling on GE81112A, finding several limitations but observing that GE81112 could be a potential target of structure/function optimization efforts (15). Such programs would greatly benefit from improved structural data (7, 9), describing the interaction of GE81112 with its ribosomal target. Accordingly, we have determined the structure of GE81112 bound to a functional bacterial 30S initiation complex, its *in vivo* target (8).

## RESULTS

### Structure overview

**Fig 1:**
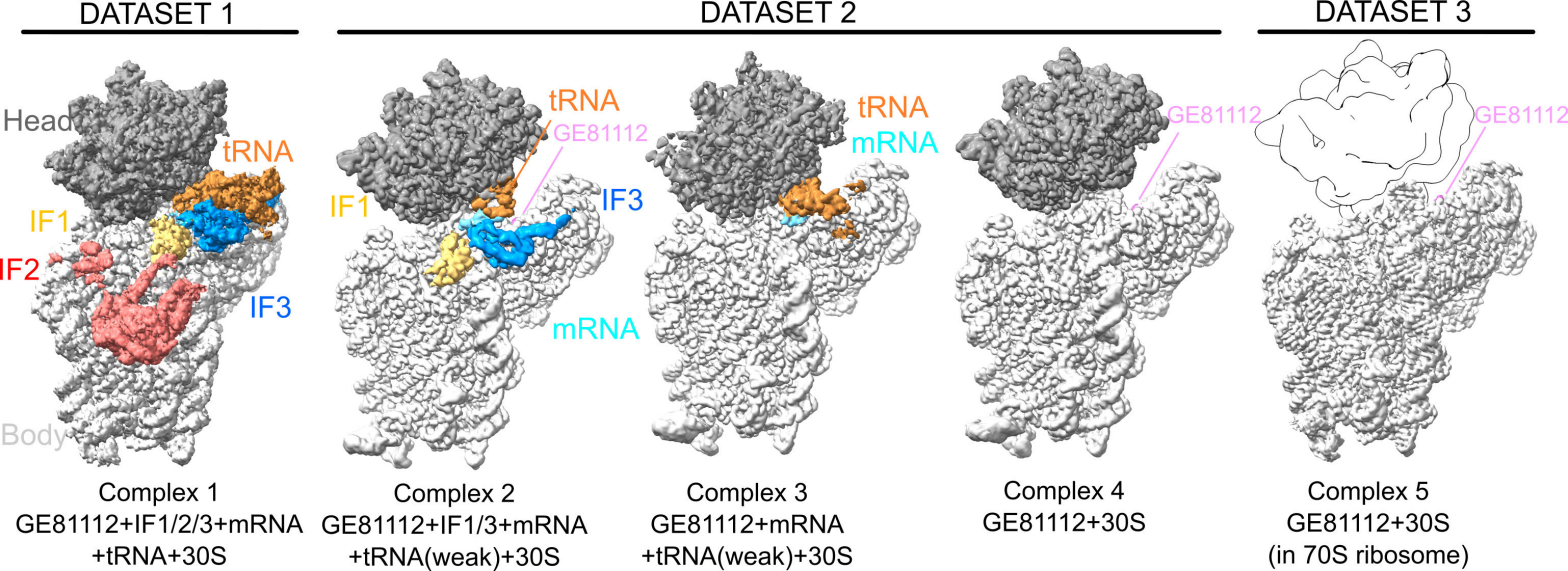
Cryo-EM structure overview. Cryo-EM maps segmented and colored to highlight density for the initiation factors, mRNA, fMet–tRNA, GE81112, and 30S head/body domains. The cryo-EM map in data set 1 results from a refinement of the entire 30S subunit. Data set 2 from a multibody refinement where the head and body regions were refined separately, and data set 3 from a refinement of the 30S body region after density corresponding to the 30S head and 50S subunit was subtracted. Maps for complexes 1–4 are sharpened with deepEMhancer (16). The maps were segmented using masks generated from the models. Density consistent with the likely presence of GE81112 is seen in all maps but obscured by the decoding arm of the fMet–tRNA when present and, therefore, is not labeled in complexes 1 and 3. To compare the quality of the density attributed to GE81112 in all complexes, the cryo-EM map surrounding the GE81112 pocket is shown from a better view in Fig. S4. In data set 3, a trace of the 30S head (based on a PDB model) is shown for clarity. For illustration, the cryo-EM maps were segmented, colored distinctly, and contoured at the following approximate levels (absolute units [abs]/standard deviations [SD]): complex 1: 0.0074/0.2; complex 2 body: 0.024/0.9; complex 2 head: 0.016/0.9; complex t3 body: 0.0239/0.9; complex 3 head: 0.0194/1; complex 4 body: 0.0174/0.7; complex 4 head: 0.0196/1.1; and complex 5 body: 0.0501/6.

To understand the interaction of GE81112 with the *Escherichia coli* ribosome, *in vitro* reactions mimicking the initiation process (*E. coli* 30S subunits, fMet–tRNA, mRNA, IF1, IF2, and IF3) were set up in the presence of GE81112 (see Materials and Methods). The reaction conditions were established in previous FRET and cryo-EM experiments, which showed that GE81112 traps the 30S in a preinitiation state (pre*IC*) (7, 9). Under these conditions, two independent samples were prepared and used in single-particle cryo-EM experiments (Fig. S1 and S2). The data set obtained from the first sample (Fig. 1, Data set 1) showed the density for all three initiation factors, albeit at a relatively low resolution (3.8 Å global resolution, complex 1: 30S + GE81112 + fMet–tRNA + IF1/IF2/IF3, Fig. S1). In comparison, the cryo-EM maps from the second sample had a much weaker density for IF2 but showed higher resolution (Fig. S2, Data set 2). This data set was classified into three well-defined sub-populations (Fig. 1) referred to as complex 2: 30S + GE81112 + fMet–tRNA(weak) + IF1/IF3 (3.3 Å consensus refinement; 3.2 Å Body; 3.2 Å head), complex 3: 30S + GE81112 + fMet–tRNA (3.3 Å consensus refinement; 3.3 Å body; 3.7 Å head), and complex 4: 30S + GE81112 (weak fMet-tRNA; 3.1 Å consensus; 3.1 Å body; 3.1 Å head). In complex 1, where IF3 and fMet-tRNA are present, the C-terminal domain of IF3 (IF3–CTD) is seen bound to the top of h44 (pos1), and the fMet-tRNA is in a pre-accommodated position when compared with the initiation complexes of Hussain et al. (Fig. S6) (5, 6). This indicates the initiation reaction was stalled in an early initiation state, suggesting that GE81112 is actively stalling the assembly process as expected (7). In complexes 1–4, lower local resolution (Fig. S1 and S2) was observed for the fMet–tRNA and initiation factors, indicating that in the pre*IC*, these elements are compositionally or conformationally dynamic. This compositional and conformational heterogeneity is consistent with the previous cryo-EM studies on the 30SIC, which show multiple states and large-scale movements, particularly in the IF3–CTD (6). For discussing the position of the IFs/fMet–tRNA and mRNA, we refer primarily to complex 1 as in data set 1, the cryoEM map for the head region was interpretable without needing multibody refinements; data set 2 required the use of multibody refinements and the regions at the interface of the body, particularly the mRNA/fMet–tRNA, are difficult to interpret. Overall, the resolution of the cryo-EM maps was sufficient to model the components of the 30S subunit at a residue level. In contrast, the lower resolution (Fig. S1 and S2) in the density of the initiation factors allowed for accurate modeling of the backbone at the secondary structure level.

In addition to the initiation complexes, a third sample (Fig. 1, Data set 3) was prepared that included only GE81112 bound to a 70S ribosomal particle (Fig. S3) to determine a high-resolution model for the GE81112 binding pocket. Single-particle cryo-EM on this complex yielded a final cryoEM map for the 30S subunit body with an overall resolution of 2.1 Å (consensus 70S map: 1.9 Å; Fig. 1). This high-quality map allowed the accurate placement of GE81112 in the map and an analysis of its interaction with the 30S subunit. Note this complex was prepared on a 70S ribosome to facilitate improved resolution; it is not meant to imply GE81112 targets 70S functions as, in fact, prior characterization indicates GE81112 is an initiation targeting antibiotic (8).

### GE81112 binds to a pocket formed by h23, h24, and h45 in the mRNA channel

**Fig 2:**
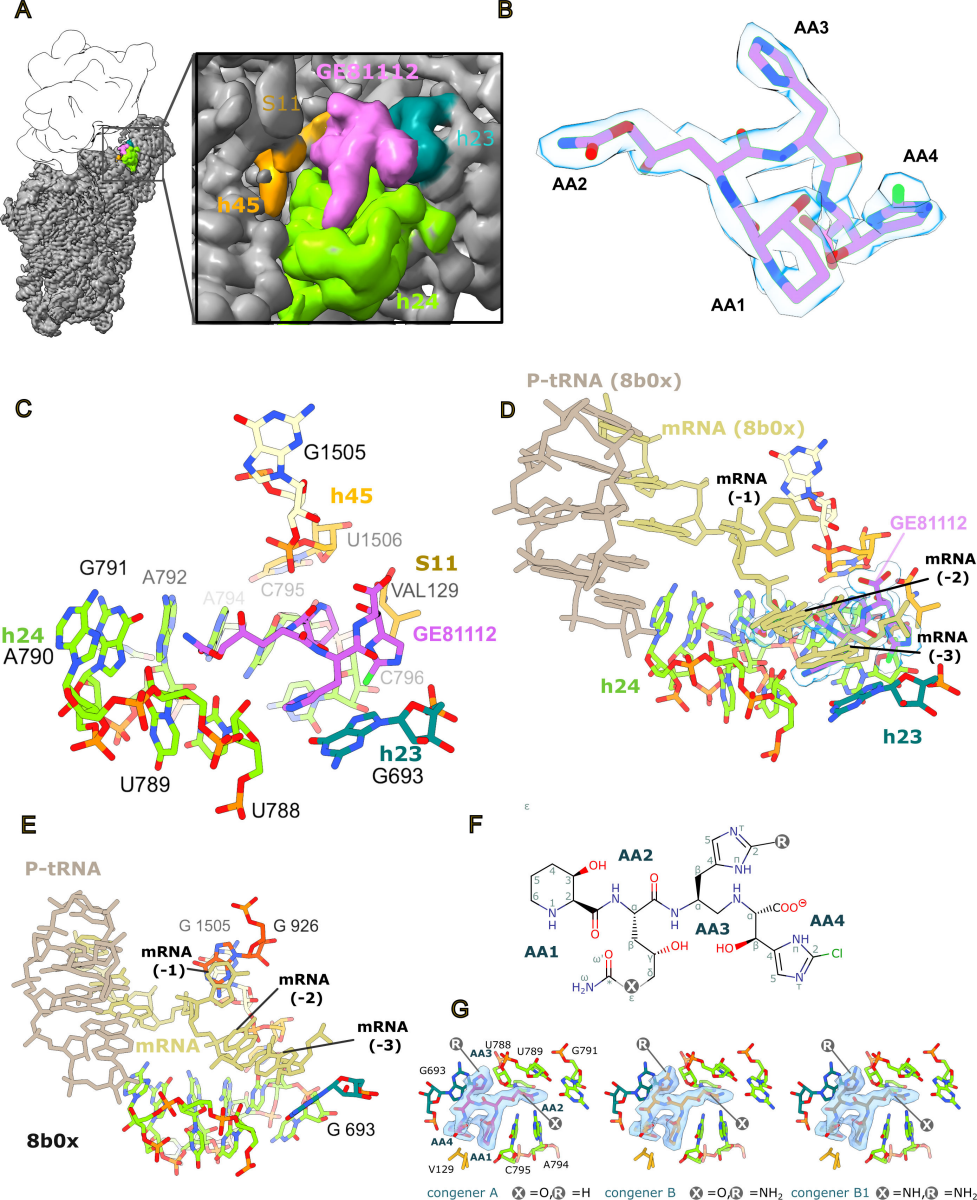
GE81112 binding site. (**A**) Overview of GE81112 binding site on 30S subunit (complex 5, unsharpened; approx. contour level: 0.06/7.2 [abs/SD]). (**B**) GE81112 (congener GE81112A) fit to the sharpened complex 5 (approx. contour level: 0.167/9 (abs/SD)) cryo-EM map. (**C**) The GE81112 binding pocket overlaps with the mRNA channel of the E-site and is formed by 16S rRNA h24, h23, and h45, and the ribosomal protein (r-protein) S11. (**D**) Aligning the GE81112 model (complex 5, GE81112 is rendered as surface) with a PDB model for a 70S ribosome bound by P-tRNA and mRNA (PDB 8b0x) shows a clash between GE81112 and the mRNA upstream of the start codon (position −2 and −3 at the mRNA E-site). (**E**) The E-site mRNA is shown on the 8b0x structure in the absence of GE81112, illustrating how the mRNA nucleotides are positioned relative to 16S rRNA nucleotides. For example, in this structure, the −1 nucleotide is stacked on G926, and the −3 nucleotide is positioned over G693. (**F**) 2D chemical structure of GE81112 with the positions that are different in the three congeners marked with an R and an X. The identities of R and X are given in panel F. (**G**) The three congeners (labeled in the figure panels) are shown inside the cryoEM density (complex 5; sharpened; approx. contour level: 0.108/6 [abs/SD]).

Previously, using X-ray crystallography and *Thermus thermophilus* 30S ribosome, we observed density consistent with GE81112 being bound close to the bent ASL of the P-site tRNA, represented in the crystal by helix 6 (h6) of a symmetry-related 30S subunit (9). This binding site is distinct from the one we observe here, using cryo-EM, on bona fide *in vitro* assembled *E. coli* 30S initiation complexes and on *E. coli* 70S ribosomes. Here, GE81112 is seen bound in a pocket that overlaps with the E-site mRNA, formed by the groove of h24 (U788–G791, A794–C796), h23 (G693), h45 (U1506), and the CTD of r-protein S11 (Fig. 2A through C). As seen in Fig. 2B, the cryo-EM density accommodates all atoms of GE81112, and in particular, the high-resolution (2.09 Å) complex five map, when sharpened, allows the orientation of the 3-hydroxy-L-pipecolic acid ring to be reliably determined; in complexes 1–4, this was ambiguous. Moreover, as mentioned, GE81112 was purified from *Streptomyces spp*. (as used in our experiments) is a mixture of three congeners, termed GE81112A, GE81112B, and GE81112B1, with slight chemical differences in AA2 and AA3 where, as depicted in Fig. 2F, either an oxygen or an NH group is present in AA2 at position ε, whereas a hydrogen or an NH2 group is present at position two in AA3. In this respect, although the complex five cryo-EM map has a high local resolution in the GE81112-binding pocket (Fig. S3), it does not indicate the presence of a specific congener, considering we use a mixture of all three to prepare the complex. For example, the map suggests the presence of an NH2 group at position R, present only in congeners B and B1 (Fig. 2G), but it does not exclude the presence of congener A (hydrogen at position R), so that we cannot exclude that the density is generated by a mixture of the congeners. Moreover, biochemical experiments using either the partially purified natural congeners (10) or chemically synthesized GE81112 congeners A and B1 both show antimicrobial activity, and SAR studies indicate that the NH2 group present at position two in AA3 is not essential (11, 12, 15). For these reasons and for simplicity, we have modeled the GE81112A congener and discussed it below. In all cryo-EM maps from the three independent data sets, GE81112 is observed in the same position, such that a comparison of the binding pocket residues yields RMSD values between 0.924 and 1.138 (297 atom pairs; Fig. S5). This indicates that the presence of fMet–tRNA and initiation factors has little effect on the conformation of the binding pocket. In the high-resolution complex 5 structure, we do notice some evidence for an alternative conformation in U793 in h24 (Fig. S6D), which is not seen in complexes 1–4. This could be due to the lower map resolution in complexes 1–4 or related to the model arising from a 70S ribosome (complex 5) rather than a 30S initiation complex (complexes 1–4). We note this, however, as chemical probing (17) indicates a connection between IF3 and U793, and this alternative conformation could be important for stalling IF3 in position 1 in the presence of GE81112.

As noted above (Fig. 2C), the GE81112 binding site involves only the rRNA (h23, h24, and h45) and r-protein S11 and does not involve any residues from the initiation factors, as seen in complexes 1 and 2. Similarly, it does not contact the fMet-tRNA. Note that in our cryo-EM maps (complexes 1–4), only mRNA nucleotides involved in base-pairing with the fMet–tRNA in the P-site show strong density. In the lower-resolution complex 1 map, there is density with a low local resolution that suggests the mRNA (−1 position) approaches GE81112 such that the mRNA backbone (−1 position) is between G926 and GE81112 (Fig. S6C). G926 is a universally conserved nucleotide that is part of hinge 1, a motif involved in 30S head movement (18). To better understand if GE81112 could potentially interfere with the mRNA outside of the P-site, we aligned a model of a 70S ribosome bound by a peptidyl-tRNA (P-tRNA) and mRNA (PDB 8b0x [19]) to the complex 5 model. As seen in Fig. 2D and E, nucleotides in the mRNA upstream of the start codon, −2 and −3 (E-site), clash with GE81112, suggesting that GE81112 interferes with the placement of the mRNA. Interfering with the placement of upstream mRNA nucleotides −2 and −3 (as seen in complexes 1–4) agrees with a previous study showing that GE81112 has only a marginal effect on the binding kinetics of model mRNAs but does affect hydroxyl radical cleavage of the mRNA, particularly upstream of the start codon (9). We propose that GE81112 prevents proper mRNA accommodation by binding in the E-site portion of the mRNA channel; specifically, we observe that AA3 of GE81112 stacks on G693, a position that is generally occupied by the −3 mRNA nucleotide (20) (Fig. 2D and E). As seen in Fig. S6C, the cryo-EM map suggests the backbone of the −1 nucleotide is positioned near G926. The importance of G926 to the mechanism of action of GE81112 is supported by the previous observation that mutations in G926 reduce the sensitivity of *in vitro* mRNA translation experiments to GE81112 (21).

### Interaction of GE81112 with the 30S subunit

**Fig 3:**
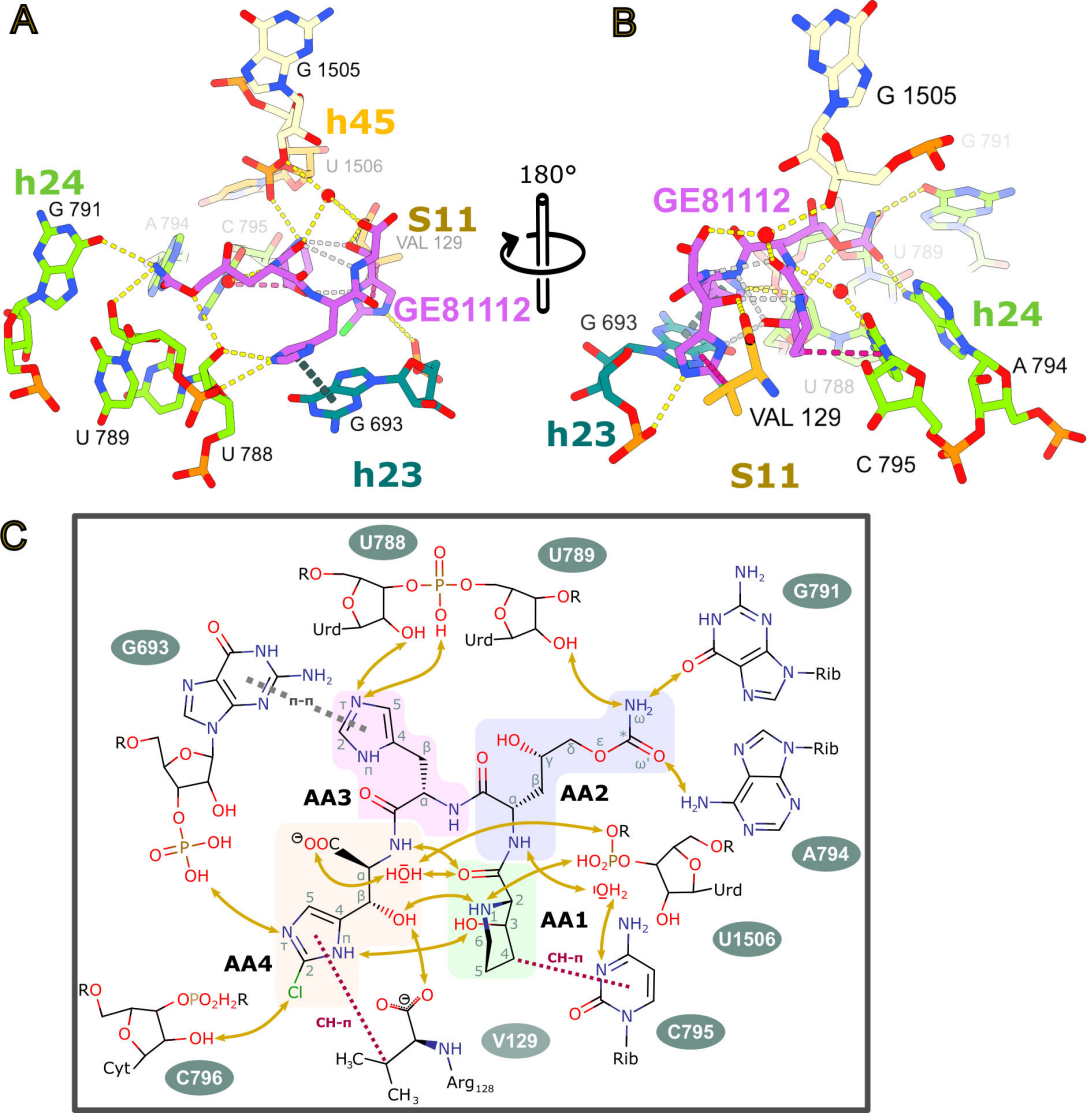
Interaction of GE81112 with the 30S binding pocket. (**A, B**) Two views of the GE81112-binding pocket highlighting interactions inferred from geometric constraints. Intermolecular hydrogen bonds are yellow, intramolecular hydrogen bonds are gray, CH-π interactions are crimson, and π stacking interactions are grey-blue. (**C**) The interactions of GE81112 with elements of its binding pocket are summarized in a 2D schematic representation. The amino acid monomers are highlighted: 3-hydroxy-L-pipecolic acid (AA1, green), 4-hydroxy-L-citrulline (AA2, violet), O-carbamoyl-α-amino-dihydroxyvaleric acid, 2-amino-L-histidine (AA3, pink), and β-hydroxy-2-chloro-L-histidine (AA4, cream).

Consistent with the hydrophilic nature of GE81112, it makes extensive hydrogen bonds with the 30S subunit. As seen in Fig. 3, AA1-4 form an extensive hydrogen bond network (shown in yellow) with 16S rRNA nucleotides in helices: h23 (G693), h24 (U788, U789, A794, and C795), h45 (U1506), and the C-terminal carboxyl moiety of ribosomal protein S11 (Val129). Moreover, the interaction of GE81112 with the 30S involves a parallel shifted aromatic π stacking (dashed lines in gray-blue) of the imidazol ring of AA3 with the base of G693, and several CH-π interactions (colored in red/crimson), including those of the methylene moieties of the 3-hydroxy piperidine ring and the isopropyl part of valine 129 with the base of C795 and the imidazol ring of residue AA1 (Fig. 3). Ligand binding is favored by the entropic gains from the displacement of at least three structural waters as identified in the high-resolution X-ray structure of vacant *E. coli* ribosome (PDB ID 4YBB [22]). The interaction of GE81112 with h23 and h24 is consistent with the chemical probing results of Brandi et al. (8), showing that G693 is protected from kethoxal modification, whereas C795, and to a lesser extent, A792, A794, and C796 all experience changes in dimethyl sulfate reactivity.

In total, GE81112 is observed to potentially make five direct base-specific interactions (G693, G791, A794, C795, and Val129) and eight direct interactions with sequence-independent groups in the RNA and protein backbone (Fig. 3C). The high number of base-specific interactions could afford some degree of targeting GE81112 derivatives to specific bacterial strains. However, it should be noted that the rRNA bases involved are highly conserved (Fig. S7; G693: 93.52%, G791: 99.88%, A794: 99.69%, C795: 99.86%) (https://crw2-comparative-rna-web.org/nucleotide-frequency/16s-rrna-model-single-base-frequency/). These base-specific interactions also render GE81112 activity sensitive to rRNA mutations. For example, it has been shown that A794G and A794U mutations alter the *in vitro* activity (e.g., mRNA translation) of GE81112 (21). As seen in Fig. 2, these mutations would alter the interaction with AA2, as the positioning of a keto moiety (instead of the 6-amino group of A794) close to the carbamoyloxy (or carbamoylamino in the case of congener B1) moiety of AA2 would cause a repulsive interaction.

The backbone conformation of the tetrapeptide ligand in the bound state closely resembles a beta-turn of type I (23), with various polar moieties of residue AA1 and AA4 stabilizing this motif. There are four direct or water-mediated hydrogen bonds between AA1 and AA4, with several involving the 3-hydroxy group of the pipecolic acid (residue AA1) and the beta-hydroxy moiety of the chlorinated beta-hydroxyhistidine (residue AA4). This is significant as the SAR studies of Zwick et al. (12) demonstrated the importance of these groups for antimicrobial activity. If these hydrogen bonds maintain the conformation of GE81112 in solution, they would lower the entropic costs for binding to the 30S subunit, potentially explaining their importance for antimicrobial activity.

### GE81112 shares an overlapping binding site with other initiation-targeting antibiotics

**Fig 4:**
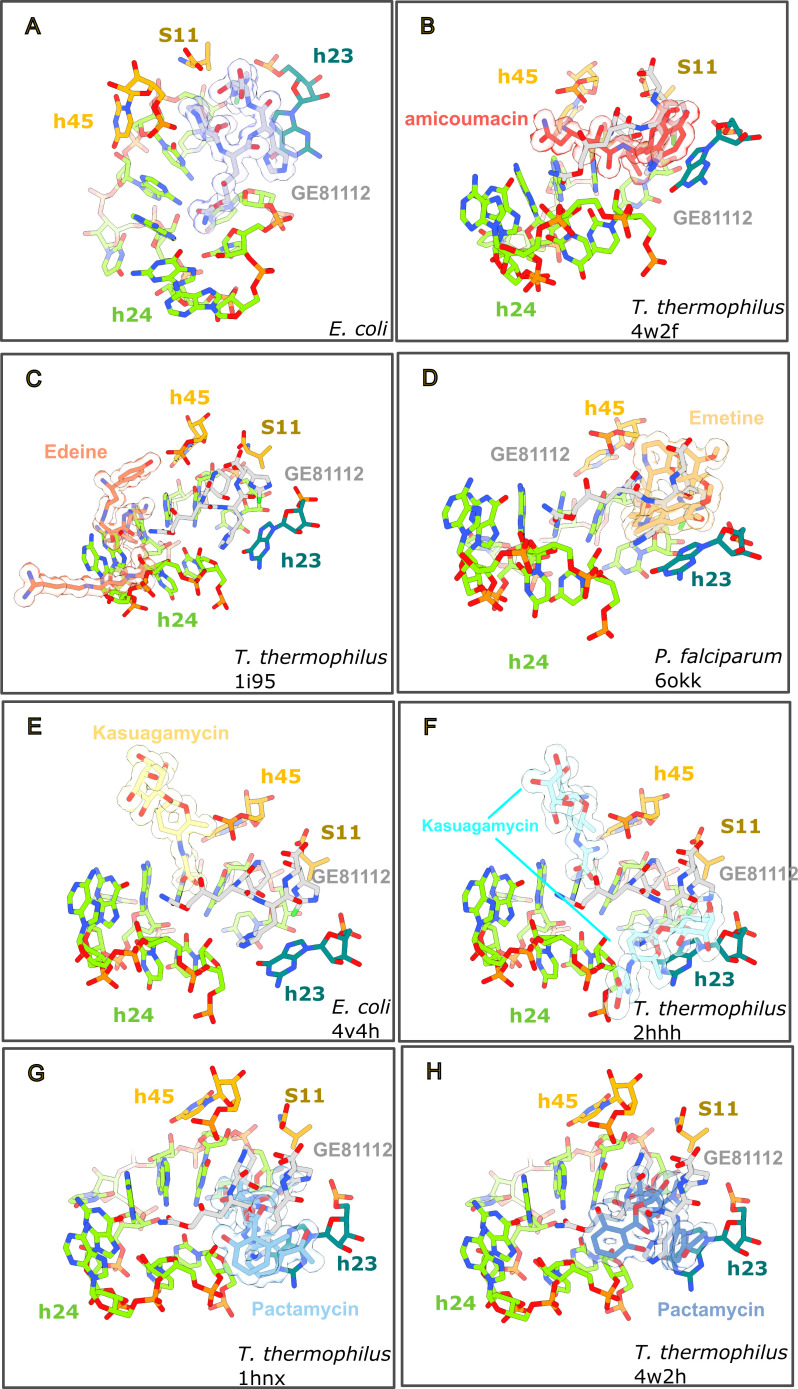
The GE81112 binding pocket is shared by other mRNA-targeting antibiotics. PDB models (**B**) 4w2f, (**C**) 1i95, (**D**) 6okk, (**E**) 4v4h, (**F**) 2hhh, (**G**) 1hnx, and (**H**) 4w2h have been aligned to the GE81112-binding pocket (**A**) using residues within 15 Å of GE81112. The antibiotics in the panels have been rendered as sticks with a transparent surface representation (calculated from PDB). The orientation in each panel was selected to highlight the relative positions of GE81112 and the other antibiotics.

Like GE81112, there are several other chemically unrelated antibiotics, amicoumacin (24), edeine (25), emetine (26), kasugamycin (27, 28), and pactamycin (24, 29) that bind within the mRNA channel. In Fig. 4, the structures of these drugs have been aligned with the GE81112 model to highlight the proximity of their binding site. Specifically, there is a substantial overlap between the binding site of GE81112 and amicoumacin (Fig. 4B), emetine (Fig. 4D), and pactamycin (Fig. 4G and H), whereas edeine and kasugamycin (Fig. 4C, E, and F) bind more outside the GE81112 pocket. Amicoumacin, for example, binds to this site but stabilizes the mRNA, affecting both translation and initiation (increased formation of erroneous 30S ICs) (24, 30). These drugs also impact mRNA function in a variety of ways. Edeine is a peptide antibiotic that blocks 30S formation by preventing P-tRNA binding (31). Emetine is an anti-protozoan drug affecting mRNA/tRNA translocation (26). Collectively, this establishes that the h24/h23 binding site, near the E-site mRNA codon, is a hotspot for disturbing mRNA function as it affects binding and recognition of codon and anticodon in both the P and A sites.

## DISCUSSION

In this study, we present five structures from three independent samples that describe the interaction between GE81112 and the *E. coli* ribosome in the context of the initiation of protein synthesis. These structures show GE81112 binds to the 30S subunit within the mRNA channel in a pocket, termed hereafter ecGE81112-binding site, formed by helices h23, h24, and h45 of 16S rRNA, as well as ribosomal protein S11 (Fig. 2). This is distinct from our previously identified site in the crystal structure of *T. thermophilus* 30S subunit (ttGE81112), which is in proximity to the distorted h6 of a symmetry-related 30S subunit, which is proposed to mimic the anticodon stem-loop of a P-tRNA (9) (Fig. S8). In the crystal, despite most of the elements that constitute the ecGE8112-binding site being present, no density was observed at this position for GE81112. In this respect, we notice that one difference between the two binding sites is represented by the C-terminal tail of S11. This is positioned differently in the *T. thermophilus* crystal, possibly because *T. thermophilus* lacks S21. Moreover, the C-terminal valine residue (V129), which is conserved among various bacterial species, is absent in *T. thermophilus* (Fig. S9A). Consequently, S11 may not contribute to GE81112 binding in *T. thermophilus* by forming CH-π interactions with GE81112 via V129, as seen in *E. coli* (Fig. 3). Regarding the ttGE81112 binding site, it is not formed in the *E. coli* cryo-EM maps obtained in this study. This may be due to the constraints imposed for the formation of the crystals, which include limiting the freedom of the ASL mimic. Also, in the crystal structure, to understand the basis of the altered h6 (ASL mimic) conformation observed, even in the Fo-Fc map, we employed a bulk solvent modeling protection approach (32, 33). In the resulting map, we could account for only part of the density generated by the ordering of the C-terminal tail of S13 (K121-K126), which is usually disordered in the crystal structures. The remaining part of the density could be explained by the presence of GE81112, although, as indicated in our previous work, it was not possible, at the resolution of the map, to distinguish the last C-terminal residues of S13 unambiguously from the amino acid moieties of GE81112, and therefore alternative arrangements are possible (9). In this respect, one possibility not considered earlier would have been to model S13 in multiple conformations, although this flexibility would be at odds with the stable conformation of the ASL mimic observed even in the Fo-fc map (9). Furthermore, considering the C-terminal residues of S13 (K121-K126) as part of the ttGE81112 binding pocket, it is relevant to note that S13 in *E. coli* is shorter, composed of just 118 amino acids (Fig. S9B), and this may also explain why this binding pocket is not observed in the *E. coli* system. Finally, considering the system explored in this study is less artificial, the resolution obtained in the cryoEM data is higher, the ecGE81112 binding site agrees best with the chemical probing data (8, 9), and the sensitivity of GE81112 *in vitro* activity to rRNA mutations (21), we consider it consistent with being the biologically active site. We also note that a similar binding site for GE81112 was reported recently by Safdari et al. (34).

As seen in Fig. 2, the map presented here nicely accommodates the chemical scaffold of GE81112, and modeling indicates the drug forms an extensive hydrogen bond network with the 16S rRNA and r-protein S11, whereas its cyclic moieties engage in π-stacking and CH-π interactions, particularly with G693 and C795 (Fig. 3). Notably, the cryo-EM analysis reveals that the initiation factors and fMet–tRNA do not directly interact with GE81112, suggesting its inhibitory mechanism stems primarily from GE81112 being positioned in the mRNA binding channel. In this position, it prevents the mRNA, the −2 and −3 positions, from being accommodated in the groove of h24; specifically, AA3 in GE81112 replaces the −3 mRNA nucleotide in stacking on G693 (20) (Fig. 2D and E). The disruption of the upstream mRNA path (−2 and −3 nucleotides, complex 1–4, Fig. S10) agrees with previous hydroxyl radical probing experiments that show GE81112 alters the interaction between the upstream nucleotides of the mRNA and 30S subunit (9). It should be noted that the solvent-exposed face of GE81112 contains carbonyl moieties that would have a repulsive effect on the backbone of the mRNA, contributing to the −2 and −3 nucleotides being disordered in our maps. The −1 mRNA nucleotide is only partially ordered in our complex C map; there appears to be density for the backbone but not the nucleobase (Fig. S1F and S6C). Importantly, the density suggests the −1 nucleotide of the mRNA is positioned near G926 (Fig. S1E and S6C), a universally conserved bulged base in the h28 (the neck) that Mohan et al. propose is the point (hinge 1) that the head pivots around during translocation (18). The nodding/swiveling movement of the head has been shown by Lopez et al. and Hussain et al. (6, 7) to alter the configuration of the P-site and reposition the codon-anticodon complex during the conversion of early pre*IC* complex to 30S IC complexes, where the fMet–tRNA moves from an unaccommodated to an accommodated state. We hypothesize that GE81112 could disfavor correct head rotation by influencing hinge one via G926. There are two routes for GE81112 to influence G926. The first would be via the −1 mRNA nucleotide; namely, in the complex C structure, the E-site portion of the mRNA is largely disordered and does not make defined interactions, for example, stacking with G926 as seen in other structures (20, 35). The second route could exploit the interaction of GE81112 with the phosphate connecting G1505 and U1506 (Fig. 3) because G1505, in fact, stacks on G926 from the other face. A role of G926 in GE81112 activity is suggested by the work of Maio et al., where mutations in G926 reduce the sensitivity of *in vitro* mRNA translation experiments to GE81112 (21).

One interesting aspect of the GE81112 model is the close proximity of AA1 and AA4, which is maintained by an extensive network of intramolecular hydrogen bonds (Fig. 3B). This network includes the 3-hydroxy group of the pipecolic acid (residue AA1) and the β-hydroxy moiety of the chlorinated β-hydroxyhistidine (residue AA4), which Zwick et al. (12) demonstrated to be critical for the antimicrobial activity. This close association of AA1 and AA4 could facilitate cyclizing the GE81112 scaffold. This is significant because cyclic peptide antibiotics are playing an increasing role in modern drug discovery efforts (36). Cyclic peptides can increase metabolic stability due to reduced degradation, whereas their reduced conformational flexibility can contribute entropically to increase target binding (36). A cyclic GE81112 derivative could also alter the uptake through the OPP system, potentially bypassing a significant pathway for GE81112 resistance (15, 21). The structural insights from this study provide a foundation for optimizing GE81112 derivatives and exploring their potential in combating bacterial infections.

## MATERIALS AND METHODS

### Assembly of the pre*IC*

Complexes for both data sets were prepared independently under identical conditions. *E. coli* ribosomes, ribosomal subunits, and translational factors were prepared as previously described (7). Using purified components, the 30S IC was assembled by incubating the 30S ribosomal subunits with GE81112 for 10 min at 37°C in Buffer IC (10 mM Tris–HCl [pH 7.7], 7 mM MgCl_2_, and 60 mM NH_4_CH_3_COO) prior to the addition of the mRNA construct containing a model Shine-Dalgarno sequence (AAG UUA ACA GGU AUA CAU ACU AUG UUU ACG AUU ACU ACG AUC), fMet–tRNA, GTP, and IF1, IF2, and IF3, and continuing the incubation at 37°C for an additional 10 min. Throughout this incubation, buffer conditions were kept constant, and the final factor concentrations were 1 µM 30S, 20 µM GE81112, 4 µM mRNA, 2.8 µM fMet–tRNA, 50 µM GTP, 3.2 µM IF1, 1.6 µM IF2, and 3.2 µM IF3.

### Vitrification and electron microscopy of pre*IC* complex

The 30S pre*IC* sample was vitrified using a Vitrobot (Thermo Fisher Scientific [TFS]) by diluting the reconstituted 30S IC in buffer IC containing 20 µM GE81112, 4 µM mRNA, 2.8 µM fMet–tRNA, and 50 µM GTP onto Quantifoil R2/2 grids. Automated data acquisition (EPU software, TFS) was performed at NeCEN (Netherlands Center for Electron Nanoscopy; Data set 1) and eBIC (Diamond Light Source, UK; Data set 2) with a Titan Krios microscope (FEI) at 300 kV equipped with a direct detector (Table S1). For Data set 1, 3,745 movies were collected at a pixel size of 1.086 Å and defocus ranging from 0.4 µm to 3.8 µm in two sessions. For Data set 2, 6,172 movies were collected, each containing 19 frames at a pixel size of 1.113 Å. More detailed imaging conditions are presented in Table S1.

### Vitrification and electron microscopy of 70S-GE81112 complex

The GE81112-70S complex was prepared by co-incubating for 10 min on ice 100 nM 70S ribosomes with 20 µM GE81112 in a buffer consisting of 10 mM Tris, pH 8.0, 14 mM MgAc, and 60 mM KCl. The sample was vitrified using a Vitrobot (TFS) on Quantifoil R1.2/1.3 (300 mesh) grids. Automated data acquisition (EPU software, TFS) was performed at the Basque Resource for Electron Microscopy (BREM) on a 300 kV X-FEG Krios G4 transmission electron cryo-microscope (TFS) and a Gatan K3 direct detector (Table S1). For Data set 3, 19,615 movies were collected, each fractionated into 40 frames at a pixel size of 0.8238 Å/px and a total exposure dose of 49.3 e-/A^2^.

### Single particle analysis

#### Data set 1

Motion correction was performed within RELION 3.1 (37) using the dose weighting and patch (5 × 5) options. Contrast transfer function (CTF) estimation for each aligned micrograph was performed using CTFFind (38); 232,679 projection images of 30S particles were picked using crYOLO (39). Initially, particles were rescaled and extracted with a pixel size of 3.01 Å, and the data set was cleaned using RELION 2D Classification (100 classes). Subsequently, the well-aligned particle projections (a total of 193,743) were used to generate an initial model. The projections were then refined in several cycles (using the initial model to start and removing particles from micrographs with an estimated max resolution greater than 7 Å), yielding a 5.2 Å map where the body region was visually more defined than the head region. This prompted us to utilize a multi-body refinement (head and body mask), which improved the resolution of the body region to 4.7 Å. The cryoEM map of the 30S body region was then used to initiate CTF refinement (beam tilt, anisotropic magnification, per particle defocus fitting, per micrograph astigmatism) and Bayesian polishing (37). CTF correction was applied to the polished particles to reduce their window size and speed up processing. After polishing, the map was refined to 3.7 Å; 3D classifications (30S mask and then an IF2 mask) were performed to remove poorly aligning particles, such that we finally retained a subset of 46,163 that yielded the final 3.8 Å map. Furthermore, multibody refinements or 3D classifications did not improve the map as judged visually or by resolution estimates. The FSC (Fourier shell correlation) plots for the consensus refinement, the final map, and local resolution estimates are shown in Fig. S1.

#### Data set 2

All data processing steps were performed within RELION 3.0 or 3.1 (37, 40); 6,172 movies (Fig. S2A) were imported and motion-corrected with Relion. Defocus estimation was performed with Gctf v1.06 (41), and subsequently, micrographs with defocus values between −0.5 and −3.25 µm were selected. Particles were picked with crYOLO ([39]; 662792 particles) and initially extracted at a pixel size of 2.226 Å/px. These particles were cleaned by 2D classification (100 classes; Fig. S2A), such that 397,515 particles were retained and used to generate an initial model. Further cleaning was performed with 3D classification so that 304,619 particles were kept and finally refined with a pixel size of 1.113 Å/px to generate a 3.2 Å cryoEM map. The cryo-EM map of the 30S body region was then used to initiate CTF refinement and Bayesian polishing (42). Subsequent refinement led to a 2.95 Å cryo-EM map. 3D classification (four classes, no image alignment, T = 16) was performed on these particles using a mask centered on the density of IF1, IF3, and the fMet–tRNA anticodon arm. This yielded three well-defined volumes that were subsequently refined to 3.3 Å (complex 2, 34,701 particles), 3.3 Å (complex 3, 28,111 particles), and 3.1 Å (complex 4). Finally, a multibody refinement was performed where body 1 corresponded to the 30S body and body 2 to the 30S head region. When present in the map, the best multibody refinements were obtained when the fMet–tRNA was included in the body 2 mask and the IFs in the body 1 mask. The FSC plots for the multibody maps, the final maps, and local resolution estimates are shown in Fig. S1.

#### Data set 3

All image processing steps were performed within the CryoSPARC and CryoSPARC Live software packages (43). Movies were imported to CryoSPARC and patch motion corrected (Fig. S3A), and the contrast transfer function was estimated. The micrographs were curated based on estimated resolution, defocus values, and motion parameters. Particles were selected in CryoSPARC using the blob and template picker workflow. The particles were windowed out and downsampled to a box size of 128 pixels × 128 pixels for initial cleaning using 2D classification (Fig. S3B). The best classes resembling 70S, 50S, and 30S ribosomes were selected, finally corresponding to 1,816,931 particles. These particles were used in an *ab initio* reconstruction job (six classes). Subsequently, heterogeneous refinement and 3D classification jobs (44) were used to separate the input projections into poorly aligning, 30S (29,389), 50S (190,467), and 70S (1,331,197) classes. The 70S particles were re-extracted at 512 pixels × 512 pixels and refined, yielding a cryo-EM map at 2.05 Å resolution (70S). Beam tilt correction (37) and reference-based motion correction were performed, and after a subsequent non-uniform refinement, using a 50S mask, a 70S cryoEM map at 1.85 Å resolution was obtained (1,329,758 particles under a 50S mask; Fig. S3D and E). A particle subtraction job was performed on these 50S aligned projections to generate projections containing signal from only the 30S body region of the 70S ribosome. After a non-uniform refinement, a cryo-EM map of the 30S body region at 2.09 Å was obtained (Fig. S3F and G, 1,329,758 particles). Local resolution was estimated with CryoSPARC using the non-uniform refinement half maps (45).

### Cryo-EM model building

The absolute configuration of GE81112 for describing the ligand topology was derived from Jürjens et al. (11). Starting ligand coordinates for refinement were generated using the RDkit package for cheminformatics (RDKit: Open-source cheminformatics; https://www.rdkit.org), and restraint information about its geometry was derived using eLBOW (46). The PDB structure 4YBB (22) was taken as an initial template for refinement and model building. Individual maps for the head (encompassing rRNA nucleotides C931 to G1386 and the ribosomal proteins S3, S7, S9, S10, S13, S14, and S19) and body domain (nucleotides A1 to C930 and G1387 to A1542 of the 16S RNA, and S3, S7, S9, S10, S13, S14, and S19 r proteins) were derived from cryo-EM maps obtained by the RELION multi-body refinement (low-pass filtered to the global resolution). After a preliminary rigid body refinement followed by several cycles of manual model-building using Coot (47) and real-space refinement in Phenix (48) (with secondary structure restraints and Ramachandran restraints), the non-ribosomal components of the complex were added at the final refinement whose starting coordinates were taken either from the PDB (PDBID 6UGG for tRNA) or from structure predictions employing AlphaFold2 (for the initiation factors) (49). Structural fragments of the ribosomal complexes not accounted for by the cryo-EM density, due to their absence or local disorder, were omitted from the final models. The quality of the obtained models was assessed using MolProbity as a part of the Phenix validation tools (48), and the guanidino carboxylic acid denotation issues were resolved by an in-house script. For figure preparation, ChimeraX (50) and Inkscape 1.3 were used.

## Data Availability

The raw electron microscopy movies, cryo-EM maps, and resulting atomic models are available at the EMPIAR, EMDB, and Protein Data Bank (PDB). The PDB accession codes are 9H9H, 9H9I, 9H9J, 9H9K, 9H9L, 9H9M, 9H9N, and 9H8G. The EMDB accession codes are 51964, 51965, 51966, 51967, 51968, 51969, 51970, and 51936. Raw data for Data sets 1, 2, and 3 are under EMPIAR accession codes 12517, 12458, and 12456. Data are available from the corresponding authors upon reasonable request.

## SUPPLEMENTAL MATERIAL

Supplemental material mbio.03978-24-s0001.pdf Supplemental figures and tables.

ASM does not own the copyrights to Supplemental Material that may be linked to, or accessed through, an article. The authors have granted ASM a non-exclusive, world-wide license to publish the Supplemental Material files. Please contact the corresponding author directly for reuse.
