# Supplementary material for: A binding site for the antibiotic GE81112 in the ribosomal mRNA channel
Source: mBio. 2025 Oct 24;16(11):e03978-24. doi: 10.1128/mbio.03978-24 (PMC12607687; doi:10.1128/mbio.03978-24)
Supplement: Supplemental material — Supplemental figures and tables. [file mbio.03978-24-s0001.pdf]

Supplemental Materials

A binding site for the antibiotic GE81112 in the ribosomal mRNA channel

Andreas Schedlbauer [1*,§], Xu Han [1*,2,§], Wouter van Bakel [2,§], Tatsuya Kaminishi [1*,3], Borja Ochoa-Lizarralde [1*,4], Idoia Iturrioz [1], Retina Çapuni [1*,5], Ransford Parry [6], Ronny Zegarra [6], David Gil-Carton [4,7,9], Jorge P. López-Alonso [1*,4,7], Kristina Barragan Sanz[4,7], Letizia Brandi [8], Claudio O. Gualerzi [8,**], Paola Fucini [1*,6,9,**] and Sean R. Connell [1*,2,9,**]

[1] Center for Cooperative Research in Biosciences (CIC bioGUNE), Basque Research and Technology Alliance (BRTA), Bizkaia Technology Park, Building 801A, 48160 Derio, Spain.
[2] Structural Biology of Cellular Machines Laboratory, Biobizkaia Health Research Institute, Cruces University Hospital, 48903 Barakaldo, Bizkaia, Spain.
[3] Graduate School of Medicine, Osaka University, 2-2 Yamadaoka, Suita, 565-0871 Osaka, Japan.
[4] Instituto Biofisika (UPV/EHU, CSIC), University of the Basque Country, 48940, Leioa, Spain.
[5] Department of Technology and Business, Western Balkans University, Highway Tiranë-Durrës, KM 7, Kashar, 1001 Tirana, Albania
[6] Research Centre for Experimental Marine Biology and Biotechnology, Plentzia Marine Station of the University of the Basque Country (PiE-UPV/EHU), Areatza Bidea, 48620 Plentzia, Basque Country, Spain.
[7] Basque Resource for Electron Microscopy, 48940, Leioa, Spain.
[8] Laboratory of Genetics, Department of Biosciences and Veterinary Medicine, University of Camerino, 62032 Camerino, Italy.
[9] IKERBASQUE, Basque Foundation for Science, 48011 Bilbao, Spain.

* Former address in the early stage of the project: Center for Cooperative Research in Biosciences (CIC bioGUNE).
** To whom correspondence should be addressed.

TABLE OF CONTENTS

1. Supplementary Tables

Supplemental Table S1: EM Data Collection and Image Processing

Supplemental Table S2: Model Validation Statistics for State 1

Supplemental Table S3: Model Validation Statistics for State 2

Supplemental Table S4: Model Validation Statistics for State 3

Supplemental Table S5: Model Validation Statistics for State 4

Supplemental Table S6: Model Validation Statistics for State 5

2. Supplementary Figures

Supplemental Figure S1: Cryo-EM Processing Results for Dataset 1.

Supplemental Figure S2: Cryo-EM Processing Results for Dataset 2.

Supplemental Figure S3: Cryo-EM Processing Results for Dataset 3.

Supplemental Figure S4: Cryo-EM density of the GE81112 pocket in Complexes 1-5

Supplemental Figure S5: Comparison of the GE81112 pocket in Complexes 1-5.

Supplemental Figure S6: The initiation complex 1 is in a pre*IC* state.

Supplemental Figure S7: Conservation of the rRNA in the ecGE81112 binding site.

Supplemental Figure S8: Comparison of the binding site locations of the ttGE81112 and ecGE81112 binding site.

Supplemental Figure S9: Conservation of r-proteins S11 and S13.

Supplemental Figure S10: Key mRNA, tRNA and 30S interactions are altered or disrupted in the GE81112 pre-initiation complex.

SUPPLEMENTARY TABLES

Supplemental Table S1: EM Data Collection and Image Processing.

**Data Collection**			
Sample	***Dataset 2 30SpreIC-GE81112***	***Dataset 1 30SpreIC-GE81112***	***Dataset 3 70S-GE81112***
Facility	eBIC	NeCEN	BREM
Microscope	Titan Krios	Titan Krios	Titan Krios
Camera	Falcon 3	K2	K3
Data Collection Software	EPU (TFS)	EPU (TFS)	EPU (TFS)
Nominal Magnification (x1000)	75	130	105
Voltage (kV)	300	300	300
Calibrated Pixel Size (Å)	1.113	1.086	0.8238
Total Exposure (e^{-}/ $Å^{2}$)	44.08	42/51	49.3
Number of Frames	19	32 (session 1)<br>20 (session 2)	40
Defocus Range (µm)	-0.5 to -3.25	-0.4 to -3.8	-0.1 to -2.5
**Image Processing**			
Motion Correction Software	motioncorr	motioncorr	cryoSPARC (patch motion correction)
CTF estimation software	Gctf	CtfFind	cryoSPARC (patch ctf estimation)
Particle Section	crYOLO	crYOLO	cryoSPARC (template picker)
Micrographs Collected	6172	3745	19615
Particles Selected	451301	232679	1694913
Classification and Refinement Software	Relion	Relion	cryoSPARC
**Model Building**			
Visualisation Software	ChimeraX	ChimeraX	ChimeraX
Refinement Software	Phenix, Isolde	Phenix, Isolde	Phenix, Isolde

Supplemental Table S2. Model Validation Statistics for ***State 1***.

Complex	state 1 (30S)
**PDB**	9H9H
**EMDB**	51964
**Model composition**	
Chains	30
Non-hydrogen atoms	58837
Protein residues	3061
Nucleotides	1616
Ligands	
GE81112	1
Mg	150
K	5
Zn	1
**RMSD deviations from ideal values**	
Bond length (Å)	0.005
Bond angles (°)	0.817
**Ramachandran plot (%)**	
Favored	97.35
Allowed	2.65
Outliers	0.00
**Other structural quality metrics**	
MolProbity score	1.73
Clash score	15.33
Rotamer outliers (%)	0.0
Cb outliers (%)	0
Cis or twisted non-trans peptide planes (%)	
Cis proline	0
Twisted proline	0
**ADP (B-factors)**	
Iso/Aniso (#)	58837/0
Min/max/mean	
Protein	8.98/165.7/58.9
Nucleotide	12.46/191.06/43.93
Ligand	7.42/140.58/42.7

*Mg^{2+} ions, K^{+} ions and water O are present with chain IDs 5 and 6, and 7, respectively, ligand as residue number 1601 in chain A.

Supplemental Table S3. Model Validation Statistics for ***State 2***.

Complex	state 2 (Head)	state 2 (Body)
**PDB**	9H9I	9H9J
**EMDB**	51965	51966
**Model composition**		
Chains	13	18*
Non-hydrogen atoms	18982	34842
Protein residues	1102	1462
Nucleotides	478	1078
Ligands		
GE81112	0	1
Mg	49	93
K	0	4
Zn	1	0
**RMSD deviations from ideal values**		
Bond length (Å)	0.005	0.006
Bond angles (°)	0.765	0.891
**Ramachandran plot (%)**		
Favored	98.00	98.61
Allowed	2.00	1.39
Outliers	0.00	0.00
**Other structural quality metrics**		
MolProbity score	1.55	1.31
Clash score	10.65	5.64
Rotamer outliers (%)	0.00	0.00
Cb outliers (%)	0	0
Cis or twisted non-trans peptide planes (%)		
Cis proline	0	0
Twisted proline	0	0
**ADP (B-factors)**		
Iso/Aniso (#)	18982/0	34843/0
Min/max/mean		
Protein	30.00/157.81/81.65	8.98/96.22/32.28
Nucleotide	20.00/191.06/71.66	12.46/84.24/31.02

*Mg^{2+} ions, K^{+} ions and water O are present with chain IDs 5 and 6, and 7, respectively, ligand as residue number 1601 in chain A.

Supplemental Table S4. Model Validation Statistics for ***State 3***.

Complex	state 3 (Head)	state 3 (Body)
**PDB**	9H9K	9H9L
**EMDB**	51967	51968
**Model composition**		
Chains	13	15*
Non-hydrogen atoms	18869	33359
Protein residues	1091	1280
Nucleotides	478	1078
Ligands		
GE81112	0	1
Mg	44	92
K	0	6
Zn	1	0
**RMSD deviations from ideal values**		
Bond length (Å)	0.007	0.006
Bond angles (°)	0.942	0.907
**Ramachandran plot (%)**		
Favored	96.00	98.73
Allowed	4.00	1.27
Outliers	0.00	0.00
**Other structural quality metrics**		
MolProbity score	2.21	1.32
Clash score	28.83	5.84
Rotamer outliers (%)	0.43	0.10
Cb outliers (%)	0	0
Cis or twisted non-trans peptide planes (%)		
Cis proline	0	0
Twisted proline	0	0
**ADP (B-factors)**		
Iso/Aniso (#)	19039/0	33357/0
Min/max/mean		
Protein	45.15/165.07/82.26	8.98/44.07/24.24
Nucleotide	20.00/191.06/71.47	12.46/84.24/31.02

*Mg^{2+} ions, K^{+} ions and water O are present with chain IDs 5 and 6, and 7, respectively, ligand as residue number 1601 in chain A.

Supplemental Table S5. Model Validation Statistics for ***State 4***.

Complex	state 4 (Head)	state 4 (Body)
**PDB**	9H9M	9H9N
**EMDB**	51969	51970
**Model composition**		
Chains	11	15*
Non-hydrogen atoms	18486	33344
Protein residues	1100	1278
Nucleotides	456	1078
Water	0	0
Ligands		
GE81112	0	1
Mg	42	95
K	0	12
Zn	1	0
**RMSD deviations from ideal values**		
Bond length (Å)	0.005	0.005
Bond angles (°)	0.826	0.575
**Ramachandran plot (%)**		
Favored	98.52	98.3
Allowed	1.48	1.67
Outliers	0.00	0.00
**Other structural quality metrics**		
MolProbity score	1.46	1.49
Clash score	8.62	9.20
Rotamer outliers (%)	0.00	0.00
Cb outliers (%)	0.00	0.00
Cis or twisted non-trans peptide planes (%)		
Cis proline	0	0
Twisted proline	0	0
**ADP (B-factors)**		
Iso/Aniso (#)	18486/0	33344/0
Min/max/mean		
Protein	45.15/154.95/81.47	8.98/42.43/24.10
Nucleotide	45.08/191.06/72.27	12.46/84.24/31.02

* Zn and Mg ions are present with chain ID 3, ligand as residue number 1601 in chain A.

Supplemental Table S6. Model Validation Statistics for ***State 5***.

Complex	state 5 (Body)
**PDB**	9H8G
**EMDB**	51936
**Model composition**	
Chains	15*
Non-hydrogen atoms	33344
Protein residues	1278
Nucleotides	1078
Water	1881
Ligands	
GE81112	1
Mg	99
K	35
Zn	0
**RMSD deviations from ideal values**	
Bond length (Å)	0.007
Bond angles (°)	0.834
**Ramachandran plot (%)**	
Favored	97.93
Allowed	2.07
Outliers	0.00
**Other structural quality metrics**	
MolProbity score	1.48
Clash score	8.47
Rotamer outliers (%)	0.10
Cb outliers (%)	0.00
Cis or twisted non-trans peptide planes (%)	
Cis proline	0
Twisted proline	0
**ADP (B-factors)**	
Iso/Aniso (#)	33344/0
Min/max/mean	
Protein	8.98/42.43/24.10
Nucleotide	12.46/84.24/31.02

* Zn and Mg ions are present with chain ID 3, ligand as residue number 1601 in chain A.

SUPPLEMENTARY FIGURES

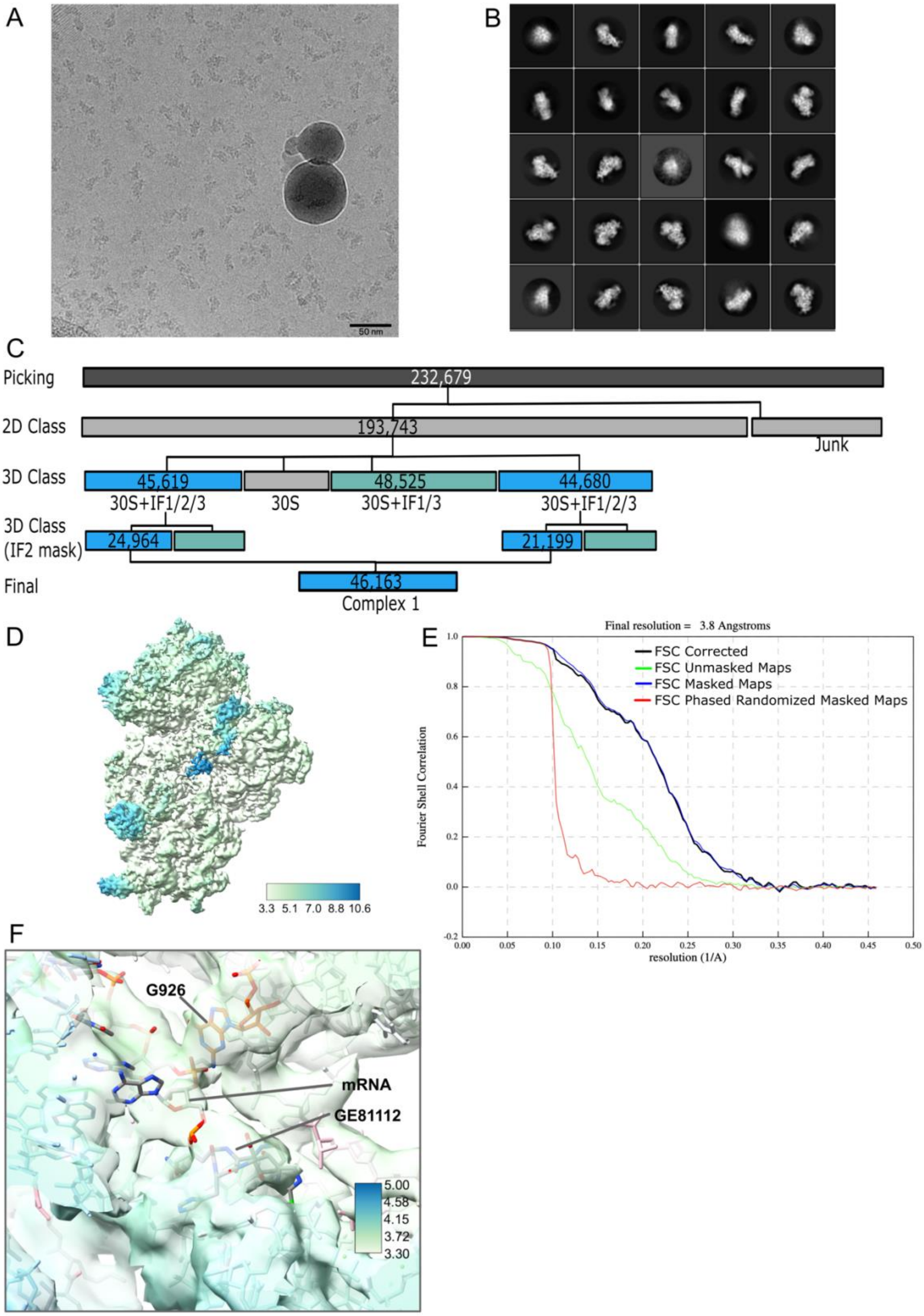

Supplemental Figure S1: Cryo-EM Processing Results for Dataset 1. (**A**) Representative cryoEM micrograph (**B**) 2D class averaging showing well-defined 30S ribosomal particles. (**C**) Workflow illustrating the classification of the dataset. (**D**) The final unsharpened cryoEM map (complex 1) is coloured according to local resolution (RELION). (**E**) The FSC curve corresponding to the cryoEM map seen in panel **C**. (**F**) The cryoEM map (unsharpened) surrounding the GE81112 binding site is coloured according to the local resolution estimate. GE81112, 16S rRNA nucleotide G926 and the -1 mRNA nucleotides are shown to highlight that in the lower-resolution cryoEM map, the mRNA approaches GE81112. Note for the local resolution colouring, the max value was set to 5 Å; therefore, the dark blue represents 5 Å or greater.

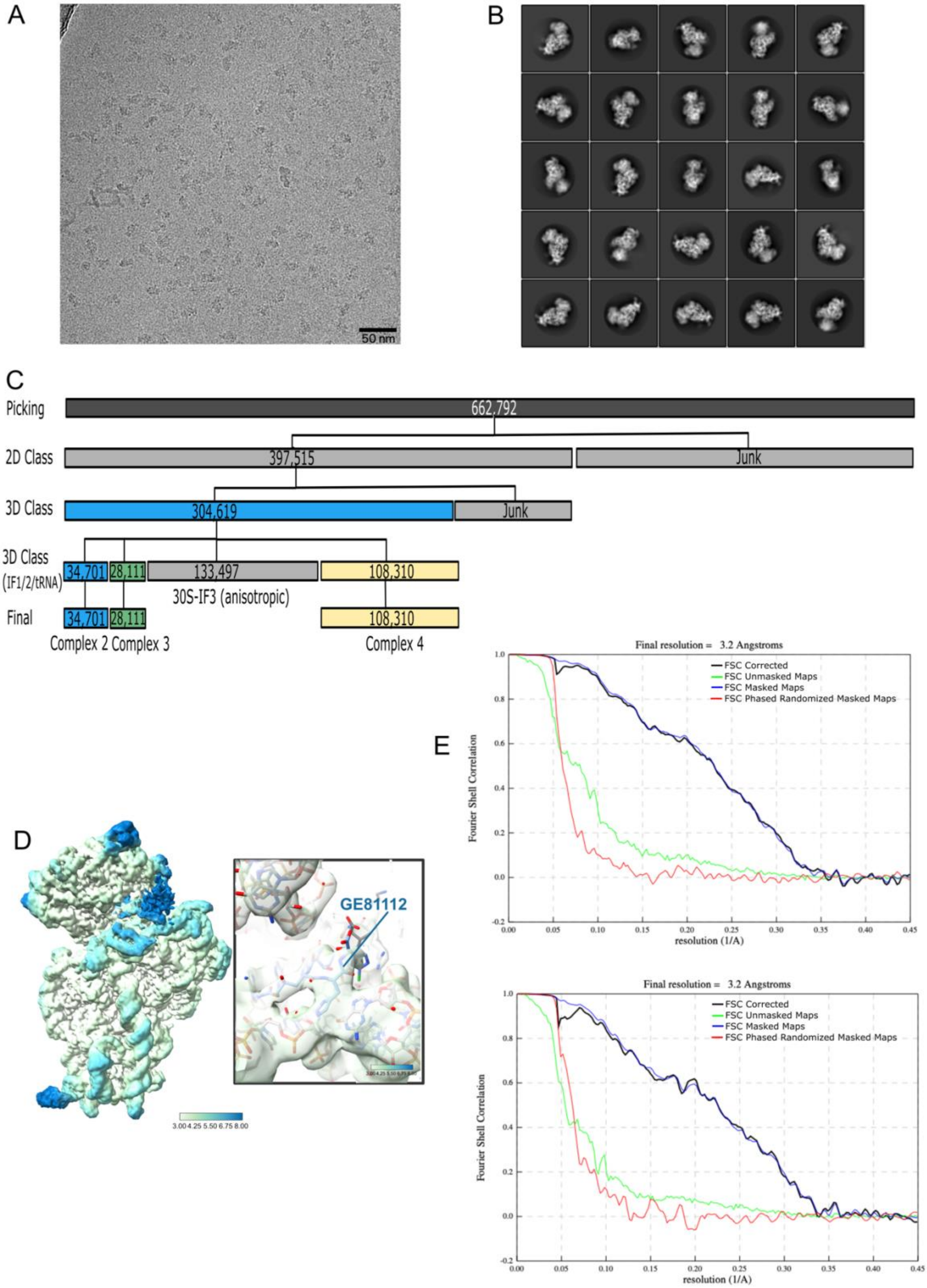

A
50 nm
B
C
Picking
662,792
2D Class
397,515
Junk
3D Class
304,619
Junk
3D Class (IF1/2/tRNA)
34,701
28,111
133,497
108,310
30S-IF3 (anisotropic)
Final
34,701
28,111
108,310
Complex 2
Complex 3
Complex 4
D
GE81112
3.00 4.25 5.50 6.75 8.00
E
Final resolution = 3.2 Angstroms
FSC Corrected
FSC Unmasked Maps
FSC Masked Maps
FSC Phased Randomized Masked Maps
Fourier Shell Correlation
resolution (1/A)
Final resolution = 3.2 Angstroms
FSC Corrected
FSC Unmasked Maps
FSC Masked Maps
FSC Phased Randomized Masked Maps
Fourier Shell Correlation
resolution (1/A)

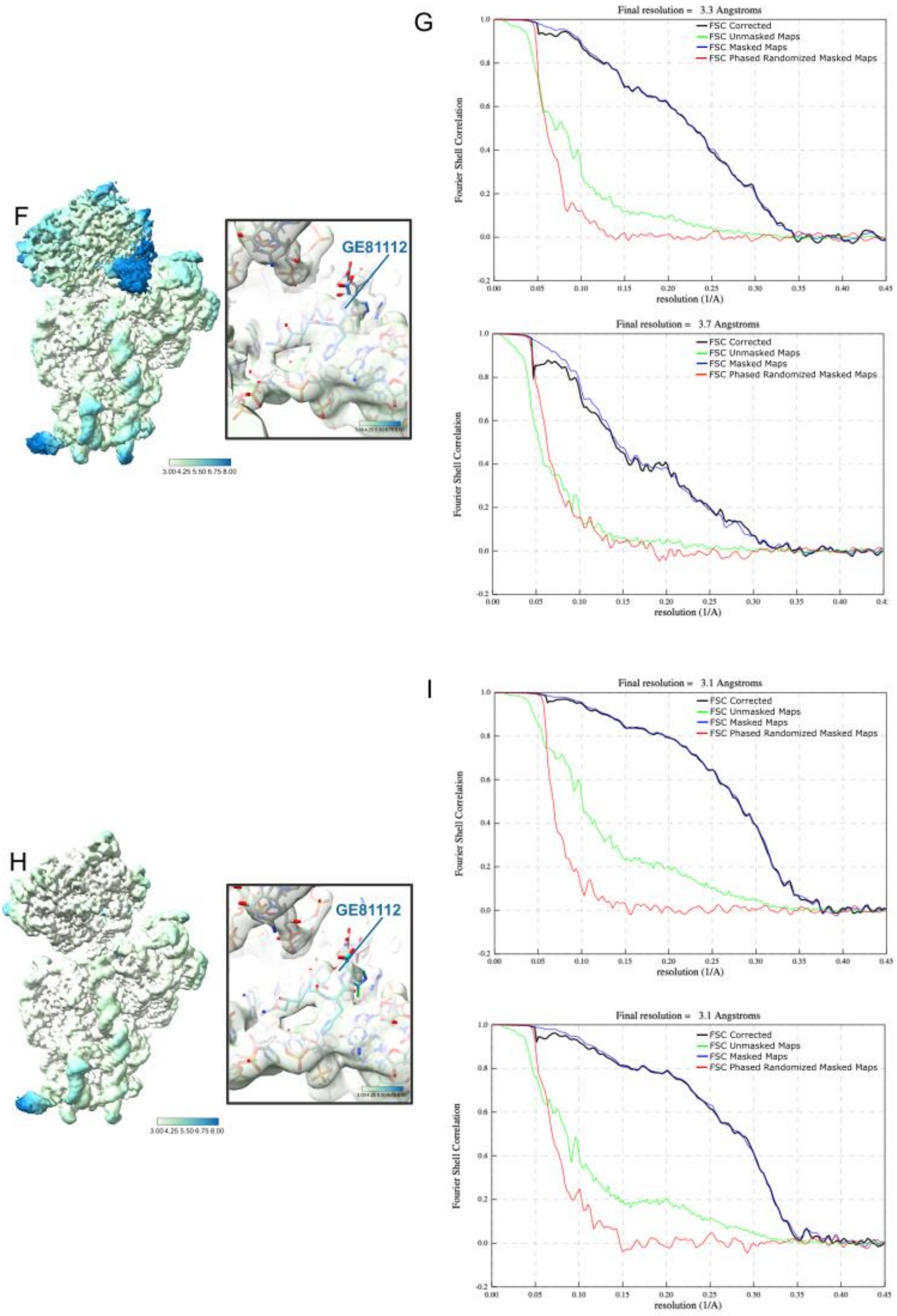

Supplemental Figure S2: Cryo-EM Processing Results for Dataset 2. (**A**) Representative cryoEM micrograph. The scalebar is 50 nm (**B**) Representative 2D classes. (**C**) Workflow illustrating the classification of the dataset. (**D**) Final multibody volumes from complex 2

colored by the local resolution (**E**) FSC curves for the multibody volumes in panel **D**; top, body; bottom, head. (**F**) Final multibody volumes from complex 3 coloured by the local resolution (**G**) FSC curves for the multibody volumes in panel **F**; top, body; bottom, head. (**H**) Final multibody volumes from complex 4 coloured by the local resolution (**I**) FSC curves for the multibody volumes in panel **H**; top, body; bottom, head. The local resolution and fall-off in the FSC curves highlight the good quality of Complex 4 (**H** and **I**), while the low local resolution and bumpy FSC curve indicates the body map (30S head) of Complex 3 (**F** and **G**) is the poorest map from dataset 2. Note for the local resolution colouring, the max value was set to 8 Å; therefore, the dark blue represents 8 Å or greater.

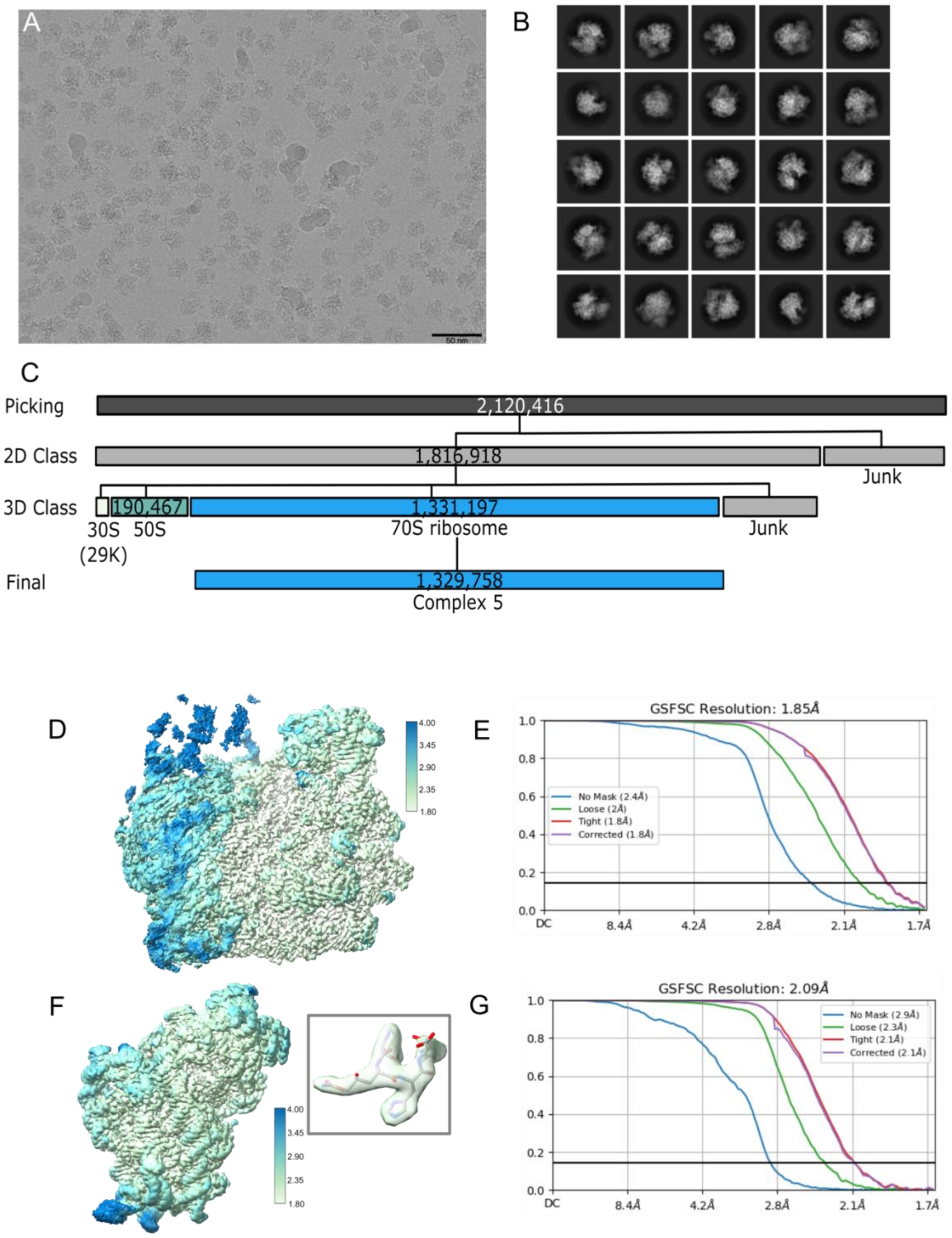

Supplemental Figure S3: Cryo-EM Processing Results for Dataset 3. (**A**) Representative cryoEM micrograph (**B**) 2D class averaging showing well-defined 70S ribosomal particles. (**C**) Workflow illustrating the classification of the dataset. (**D**) The unshapen cryoEM coloured according to local resolution (cryoSPARC) corresponds to a 70S particle refined under a mask for the 50S subunit. This volume was used in the subtraction job to yield projections of the

30S body that produced the cryoEM map seen in panel **E**. (**E**) The FSC curve corresponding to the cryoEM map seen in panel **C**. (**F**) The final cryoEM map (complex 5) corresponding to the 30S body region which harbours the GE81112 binding site (inset). The map is coloured according to the local resolution estimate (cryoSPARC) and masked to remove density for the 50S subunit that remained after subtraction. (**G**) The FSC curve corresponding to the cryoEM map seen in panel **F**. Note that for the local resolution colouring, the max value was set to 4 Å; therefore, the dark blue represents 4 Å or greater.

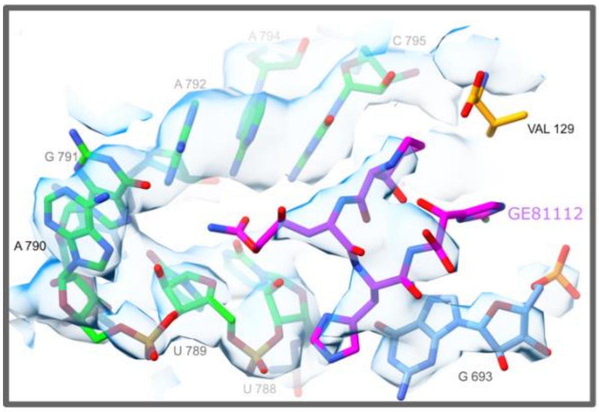

Complex 1

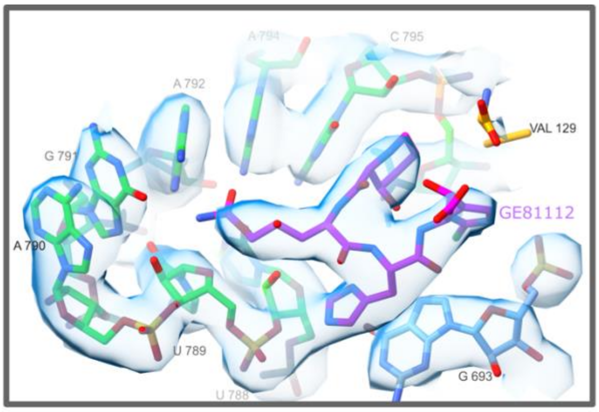

Complex 2

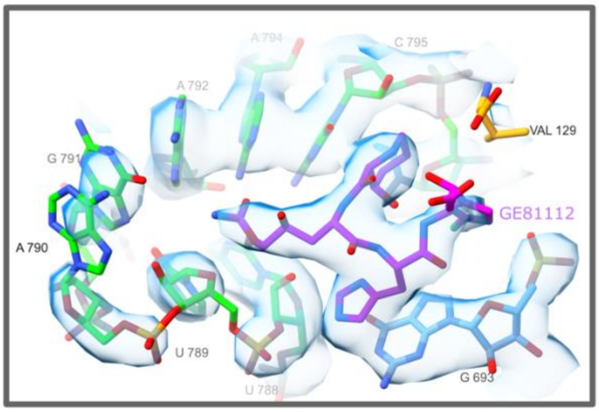

Complex 3

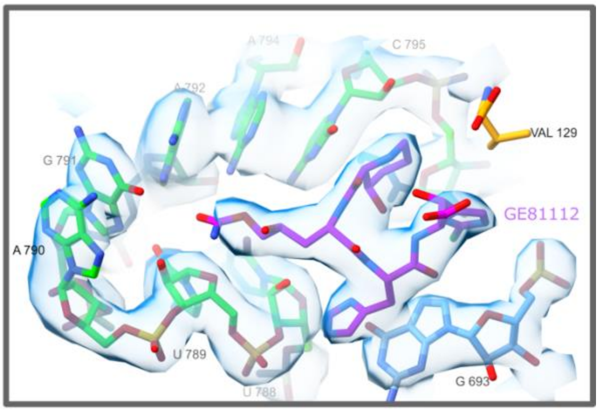

Complex 4

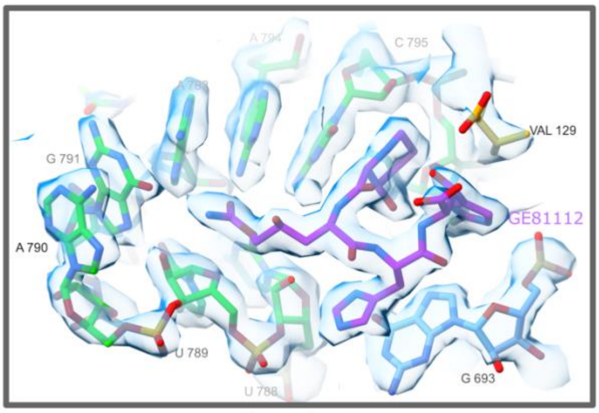

Complex 5

Supplemental Figure S4: Cryo-EM density of the GE81112 pocket in Complexes 1-5. The sharpened cryo-EM map corresponding to the GE81112 binding pocket (G81112, 16S rRNA (693,788-796) and S11 (V129)) is contoured at the following approximate levels (abs/ SD)**:** Complex 1: 0.261 / 7; Complex 2: 0.186 / 7; Complex 3: 0.183 / 7; Complex 4: 0.173 / 7; Complex 5: 0.13 / 7.

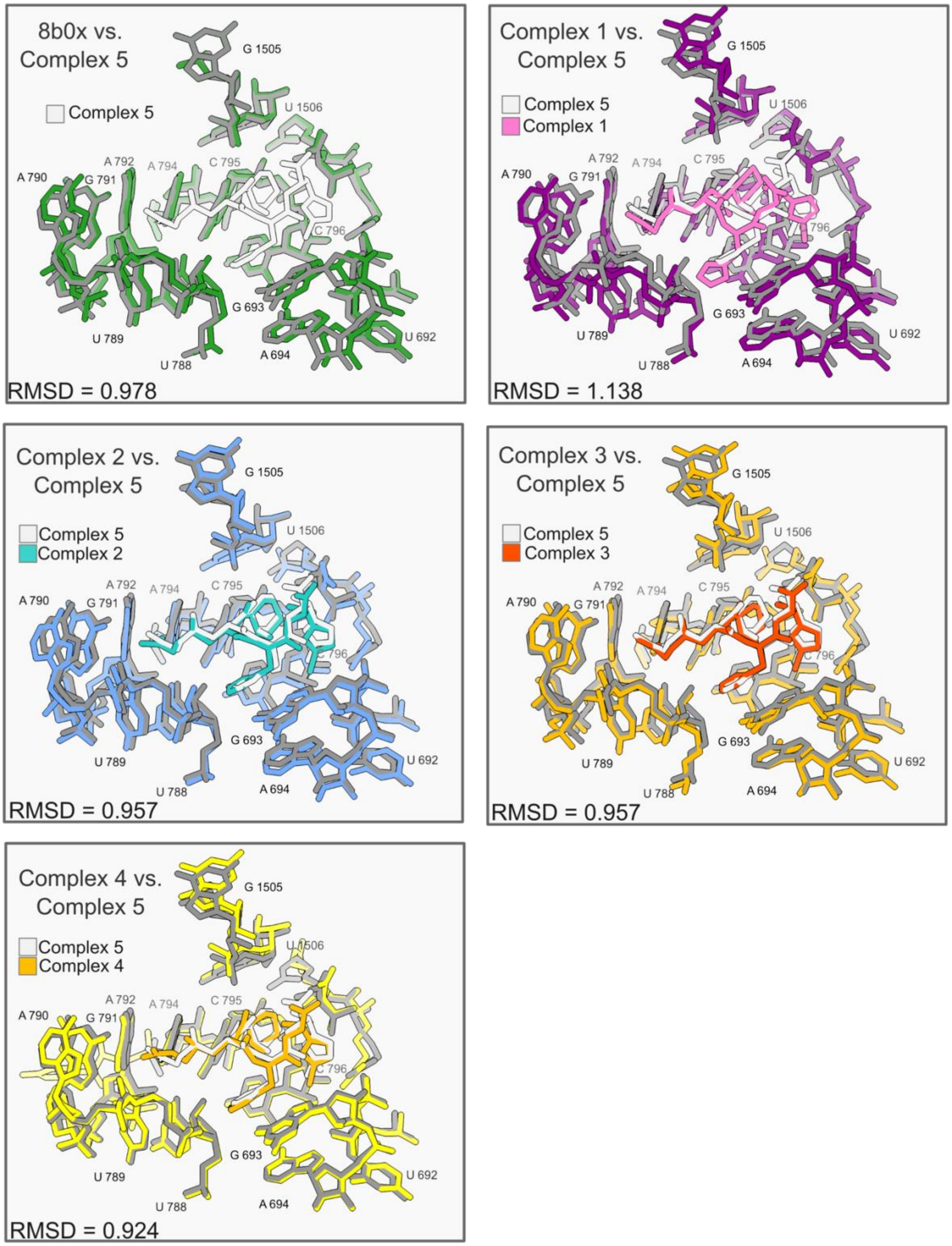

Supplemental Figure S5: Comparison of the GE81112 pocket in Complexes 1-5. The models for the five complexes and a 70S ribosome bound by a P-tRNA (8b0x; (1)) were aligned using residues within 15 Å of GE81112, and the root-mean-square deviation (RMSD) between two sets of atoms constituting the GE81112 binding pocket (16S rRNA: 692-694,788-796,1505,1506) was calculated with UCSF ChimeraX (2). Calculated values are: 0.978 (8b0x

vs complex 5); 1.138 (complex 1 vs complex 5); 0.957(complex 2 vs complex 5); 0.957(complex 3 vs complex 5); 0.924(complex 4 vs complex 5).

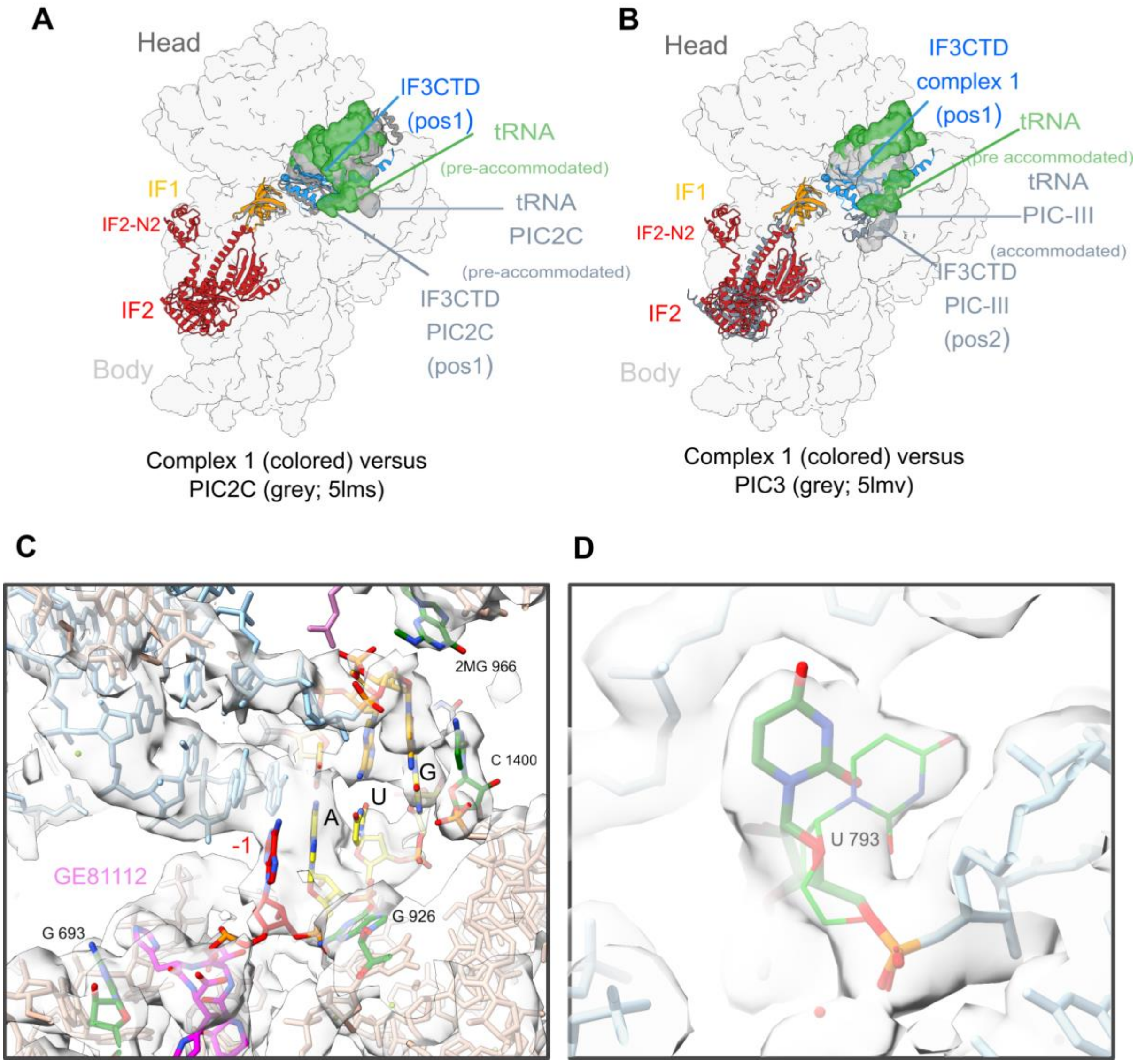

Supplemental Figure S6: The initiation complex (Complex 1) is in a pre*IC* state. The position of fMet-tRNA, IF-1(orange), IF-2(crimson), and IF-3 in complex 1 are compared to that seen in the (**A**) PIC2C (5lms) and (**B**) PIC-III (5lmv) complexes of Hussain *et al.* (3). The 30S body and head are shown as a flat surface and based on the complex 1 model. The alignment was made using 16S rRNA nucleotides in the body domain. PIC2C and PIC-III show the IF3-CTD domain in 2 different positions, pos1 and pos2. Previously, we observed the GE81112 binding is associated with IF3-CTD being bound in pos1 (4). As seen in panel **A,** the CTD of IF3 in complex I is positioned in pos1, and the fMet-tRNA is in a pre-accommodated position, indicating that GE81112 has trapped the complex in a pre*IC* state. As seen in panel (**C**), an inspection of the codon-anticodon region of complex 1 is also indicative of the fMet-tRNA being in a pre-accommodated state, for example, the density for the +1 (U) base pair is weak, and the spacing between C1400 and G966 is too far for them to interact (5 Å) although C1400 and G966 are positioned to press against the last basepair of the codon-anticodon duplex. The backbone of the -1 nucleotide shows some density, but the nucleobase is largely unaccounted for. Note we also observe density for the N2 domain of IF2 interacting near h16 (panels **A** and **B**), similar to that reported for the *Pseudomonas aeruginosa* initiation complex (5). (**D**) The high-resolution Complex 5 map (unsharpened) shows density indicative of an

alternative confirmation of U793 (main conformation green thick sticks, alternative conformation thin green sticks). The maps of Complexes 1-4 show U793 in the main conformation. All chains other than the initiation factors have been rendered as a surface. In panels A and B, Complex 1 was used to visualise the IFs and fMet-tRNA, as in Complexes 2-4, we used MultiBody refinements and treated the body + IFs as a separate body from the head +tRNA/mRNA. As such, the models of the two bodies are out of alignment with each other after the refinement and density at the interface is problematic to interpret.

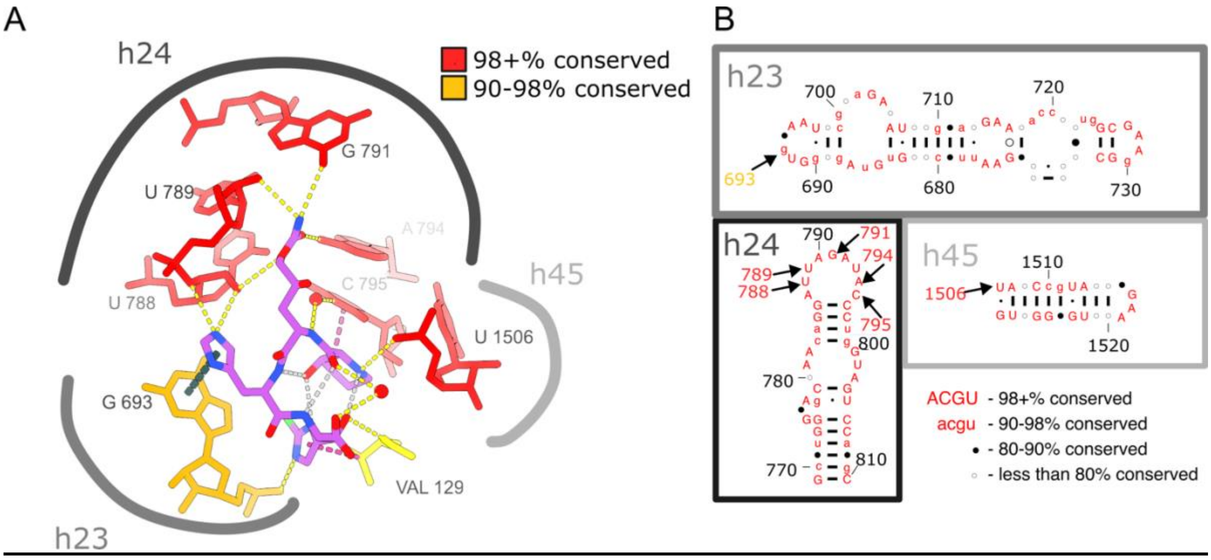

Supplemental Figure S7: Conservation of the rRNA in the ecGE81112 binding site. (**A**) rRNA nucleotides interacting with GE81112 have been colored according to their conservation using data from https://crw2-comparative-rna-web.org/nucleotide-frequency/16s-rrna-model-single-base-frequency/. (**B**) The conservation of h23, h24 and h45 is also indicated on the secondary structure maps from the website above.

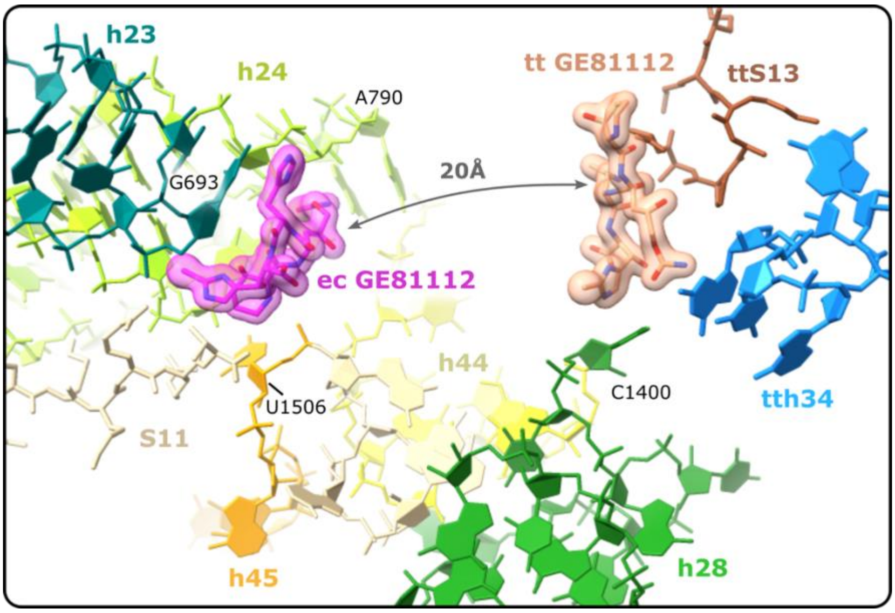

Supplemental Figure S8: Comparison of the binding site locations of the ttGE81112 and ecGE81112 binding site. The *Thermus thermophilus* (pdbID 5IWA) 30S subunit was aligned to the *E. coli* 30S ribosomal subunit (dataset 1), highlighting the relative position of GE81112 in the two structures. Helix 6 (h6) of the symmetry-related entity in the *T. thermophilus* X-ray structure, which participates in crystal contacts and mimics the P-site tRNA, is omitted in the figure for clarity. However, it would be positioned between the two binding sites.

A

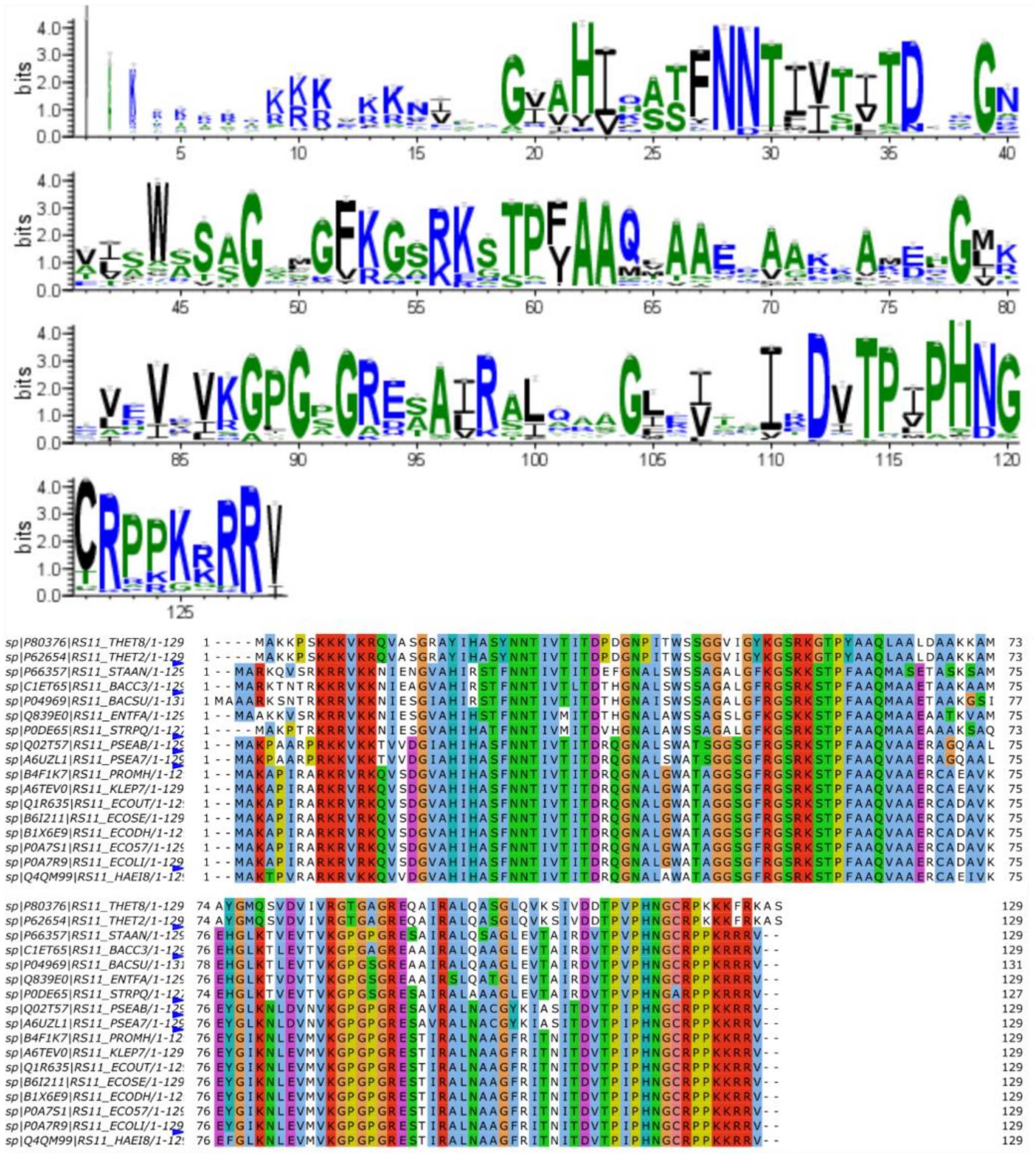

B

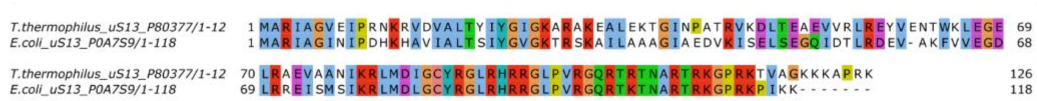

Supplemental Figure S9: Conservation of r-proteins S11 and S13. (A) The conservation of r-protein S11 is illustrated with WebLogo (top) with selected sequences aligned in below. (**B**) Clustal alignment of *T. thermophilus* (P80377) and *E. coli* (P0A7S9) r-protein S13.

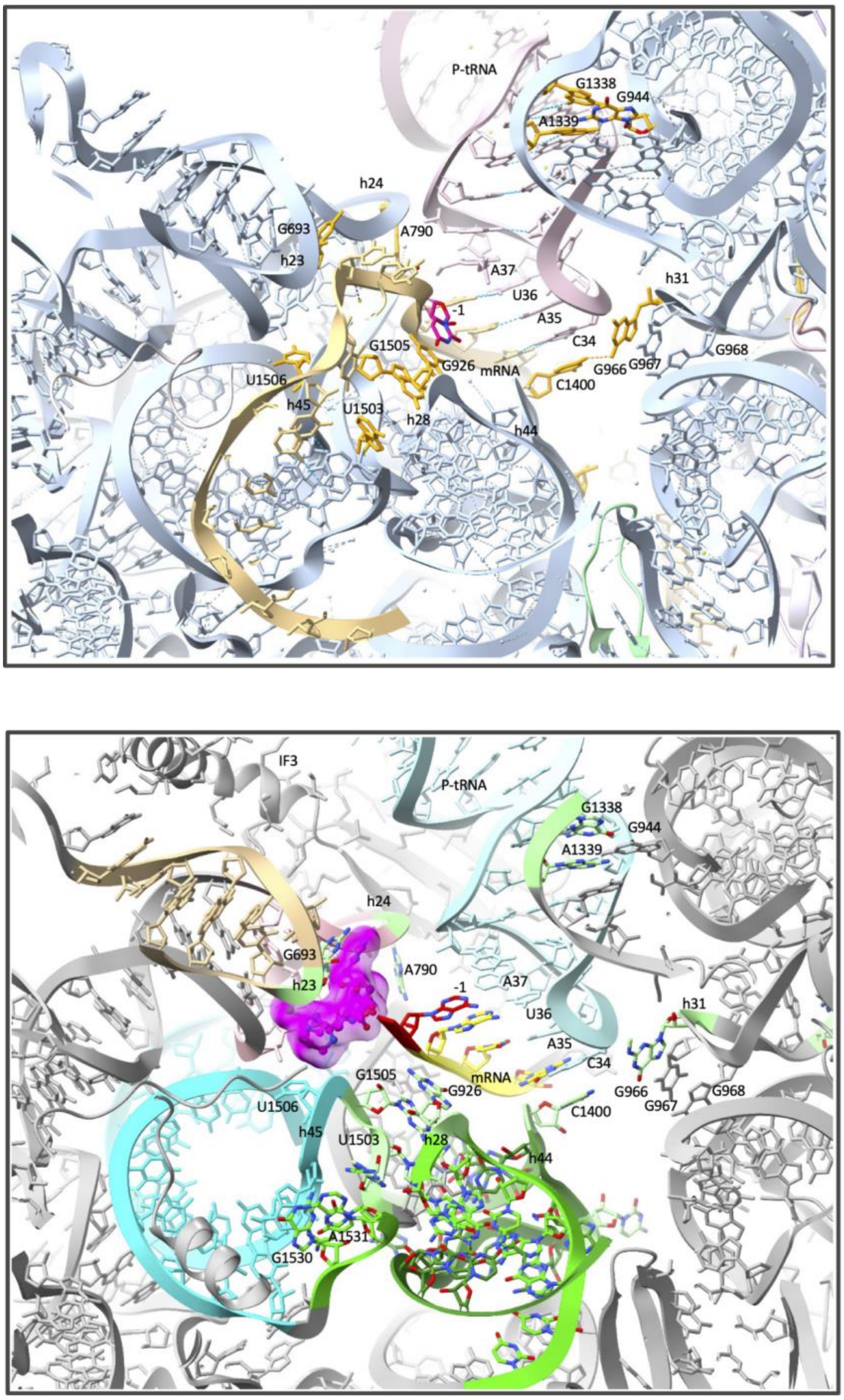

Supplemental Figure S10: Key mRNA, tRNA and 30S interactions. GE81112 (bottom panel, magenta, as observed in this study, in Complex 1), impedes the correct positioning of the mRNA (gold ribbon; top panel) in the E-site. The interactions that characterise the full accommodation of the mRNA and tRNAs, have been previously described (6–10) and are depicted in the top panel, using the *Thermus thermophilus* structure 4v6f. Interestingly, these interactions are formed with nucleotides located either at the tip/loop or in bulge positions of helices of the 30S body (i.e. G693, h23 tip (E-site mRNA and tRNA); U1503, G1505 and U1506, loop connecting h44 and h45 (E-site); C1400, h44 tip (P-site tRNA); the universally conserved nucleotides involved in the decoding, interact with both mRNA and tRNA at the A-site: A1492 and A1493 in h44 and G530, in the 530 loop of h18) and the head ((i.e. G926 in

h28 (E-site, mRNA -1); C1054, in bulge position in h34 (mRNA and tRNA A-site, decoding) and 1534, which is part of the SD-aSD helix and interacts with G929 of h28; G1338 and A1339 in the loop that connects h29 to h42, (P-site tRNA); A790, tip h24, (P-site tRNA); G966, tip h31 (P-tRNA); G926, bulge position in h28, interacts with the mRNA P-site) (6–10). Importantly, the ribosome does not interact with the mRNA at the P-site so that its positioning on this site is ensured through other interactions. In this respect, it could play a critical role the set of interactions between the ribosome and the mRNA at the E-site, represented by a strong polar hydrogen bond with the phosphate at the +1 mRNA position and a stacking interaction with the base of G926 with the mRNA in position -1 (10), depicted in magenta in the top panel. In the pre-initiation complex obtained in this study, in the presence of GE81112 (Complex 1, bottom panel), although G926 is involved in an interaction with a backbone phosphate of the mRNA, the base of the preceding mRNA nucleotide (depicted in red), does not perform a stacking interaction with G926 as it is instead base paired with A37 of the P-tRNA. Interestingly, in this complex, 3 additional bases of the mRNA are base paired with the tRNA, at position C34, A35 and U35. In this complex, the nucleotides described above, involved in the key interactions between mRNA, tRNA and the ribosome result in slightly different relative positioning, in concomitance with a different orientation of the head. In this respect, the key interaction between G966 and C1400 that indeed stabilises the closed conformation of the head of the ribosome, is not performed as the two residues result at a distance of more than 6 Å.

REFERENCES

1. Fromm SA, O'Connor KM, Purdy M, Bhatt PR, Loughran G, Atkins JF, Jomaa A, Mattei S. 2023. The translating bacterial ribosome at 1.55 Å resolution generated by cryo-EM imaging services. Nat Commun 14:1095.
2. Meng EC, Goddard TD, Pettersen EF, Couch GS, Pearson ZJ, Morris JH, Ferrin TE. 2023. UCSF ChimeraX: Tools for structure building and analysis. Protein Science 32:e4792.
3. Hussain T, Llácer JL, Wimberly BT, Kieft JS, Ramakrishnan V. 2016. Large-Scale Movements of IF3 and tRNA during Bacterial Translation Initiation. Cell 167:133-144.e13.
4. López-Alonso JP, Fabbretti A, Kaminishi T, Iturrioz I, Brandi L, Gil-Carton D, Gualerzi CO, Fucini P, Connell SR. 2017. Structure of a 30S pre-initiation complex stalled by GE81112 reveals structural parallels in bacterial and eukaryotic protein synthesis initiation pathways. Nucleic acids research 45.
5. Basu RS, Sherman MB, Gagnon MG. 2022. Compact IF2 allows initiator tRNA accommodation into the P site and gates the ribosome to elongation. Nat Commun 13:3388.
6. Hoffer ED, Hong S, Sunita S, Maehigashi T, Gonzalez RL, Whitford PC, Dunham CM. Structural insights into mRNA reading frame regulation by tRNA modification and slippery codon–anticodon pairing. eLife 9:e51898.
7. Mohan S, Donohue JP, Noller HF. 2014. Molecular mechanics of 30S subunit head rotation. Proceedings of the National Academy of Sciences 111:13325–13330.
8. Nishima W, Girodat D, Holm M, Rundlet EJ, Alejo JL, Fischer K, Blanchard SC, Sanbonmatsu KY. 2022. Hyper-swivel head domain motions are required for complete mRNA-tRNA translocation and ribosome resetting. Nucleic Acids Res 50:8302–8320.
9. Noller HF, Lancaster L, Zhou J, Mohan S. 2017. The Ribosome Moves: RNA Mechanics and Translocation. Nat Struct Mol Biol 24:1021–1027.
10. Noller HF, Donohue JP, Gutell RR. 2022. The universally conserved nucleotides of the small subunit ribosomal RNAs. RNA 28:623–644.
